# Identification of a glycolysis- and lactate-related gene signature for predicting prognosis, immune microenvironment, and drug candidates in colon adenocarcinoma

**DOI:** 10.3389/fcell.2022.971992

**Published:** 2022-08-23

**Authors:** Cong Liu, Dingwei Liu, Fangfei Wang, Jun Xie, Yang Liu, Huan Wang, Jianfang Rong, Jinliang Xie, Jinyun Wang, Rong Zeng, Feng Zhou, Jianxiang Peng, Yong Xie

**Affiliations:** ^1^ Department of Gastroenterology, Digestive Disease Hospital, The First Affiliated Hospital of Nanchang University, Nanchang, Jiangxi, China; ^2^ Gastroenterology Institute of Jiangxi Province, Nanchang, Jiangxi, China; ^3^ Key Laboratory of Digestive Diseases of Jiangxi Province, Nanchang, Jiangxi, China; ^4^ Jiangxi Clinical Research Center for Gastroenterology, Nanchang, China

**Keywords:** colon adenocarcinoma, subtypes, immune microenvironment, prognosis, drugs, glycolysis, lactate

## Abstract

**Background:** Colon adenocarcinoma (COAD), a malignant gastrointestinal tumor, has the characteristics of high mortality and poor prognosis. Even in the presence of oxygen, the Warburg effect, a major metabolic hallmark of almost all cancer cells, is characterized by increased glycolysis and lactate fermentation, which supports biosynthesis and provides energy to sustain tumor cell growth and proliferation. However, a thorough investigation into glycolysis- and lactate-related genes and their association with COAD prognosis, immune cell infiltration, and drug candidates is currently lacking.

**Methods:** COAD patient data and glycolysis- and lactate-related genes were retrieved from The Cancer Genome Atlas (TCGA) and Gene Set Enrichment Analysis (GSEA) databases, respectively. After univariate Cox regression analysis, a nonnegative matrix factorization (NMF) algorithm was used to identify glycolysis- and lactate-related molecular subtypes. Least absolute shrinkage and selection operator (LASSO) Cox regression identified twelve glycolysis- and lactate-related genes (ADTRP, ALDOB, APOBEC1, ASCL2, CEACAM7, CLCA1, CTXN1, FLNA, NAT2, OLFM4, PTPRU, and SNCG) related to prognosis. The median risk score was employed to separate patients into high- and low-risk groups. The prognostic efficacy of the glycolysis- and lactate-related gene signature was assessed using Kaplan–Meier (KM) survival and receiver operating characteristic (ROC) curve analyses. The nomogram, calibration curves, decision curve analysis (DCA), and clinical impact curve (CIC) were employed to improve the clinical applicability of the prognostic signature. Gene Ontology (GO) and Kyoto Encyclopedia of Genes and Genomes (KEGG) pathway enrichment analyses were performed on differentially expressed genes (DEGs) from the high- and low-risk groups. Using CIBERSORT, ESTIMATE, and single-sample GSEA (ssGSEA) algorithms, the quantities and types of tumor-infiltrating immune cells were assessed. The tumor mutational burden (TMB) and cytolytic (CYT) activity scores were calculated between the high- and low-risk groups. Potential small-molecule agents were identified using the Connectivity Map (cMap) database and validated by molecular docking. To verify key core gene expression levels, quantitative real-time polymerase chain reaction (qRT–PCR) assays were conducted.

**Results:** We identified four distinct molecular subtypes of COAD. Cluster 2 had the best prognosis, and clusters 1 and 3 had poor prognoses. High-risk COAD patients exhibited considerably poorer overall survival (OS) than low-risk COAD patients. The nomogram precisely predicted patient OS, with acceptable discrimination and excellent calibration. GO and KEGG pathway enrichment analyses of DEGs revealed enrichment mainly in the “glycosaminoglycan binding,” “extracellular matrix,” “pancreatic secretion,” and “focal adhesion” pathways. Patients in the low-risk group exhibited a larger infiltration of memory CD4+ T cells and dendritic cells and a better prognosis than those in the high-risk group. The chemotherapeutic agent sensitivity of patients categorized by risk score varied significantly. We predicted six potential small-molecule agents binding to the core target of the glycolysis- and lactate-related gene signature. ALDOB and APOBEC1 mRNA expression was increased in COAD tissues, whereas CLCA1 and OLFM4 mRNA expression was increased in normal tissues.

**Conclusion:** In summary, we identified molecular subtypes of COAD and developed a glycolysis- and lactate-related gene signature with significant prognostic value, which benefits COAD patients by informing more precise and effective treatment decisions.

## Introduction

Colon adenocarcinoma (COAD) is the third most prevalent malignancy and second main cause of cancer-related mortality globally, and its rapid progression, high mortality and poor prognosis have emerged as a growing public threat to human health (Keum and Giovannucci, 2019). Despite many advances in the available therapeutic strategies for COAD, including surgery, radiotherapy, chemotherapy, immunotherapy, and adjuvant therapy, improving the survival of COAD patients remains a major clinical challenge (Sveen et al., 2020; Xie et al., 2020). Consequently, an immediate need exists to identify a novel potential biomarker for prognostication to guide personalized therapy for the early management of COAD and alleviate the growing public health burden of this disease.

Substantial research has indicated that the tumor immune microenvironment is closely related to COAD (Pedrosa et al., 2019; Chen et al., 2021). To adapt to a complex immune microenvironment, tumor cells develop dynamic metabolic heterogeneity, which has been identified as a critical feature of cancer (Xiao et al., 2019). Glycolysis is one of the most common metabolic reprogramming pathways, of which the Warburg effect is the most prominent. The Warburg effect is a metabolic characteristic of most tumor cells, which display increased glycolysis to convert glucose to lactate despite the presence of sufficient oxygen (Fresquet et al., 2021). The accumulation of lactate during this process creates an acidic microenvironment that promotes the formation and progression of tumors. PPFIA4 expression attenuates glycolysis and suppresses colon cancer cell proliferation, migration and invasion *via* PFKFB3/ENO2 signaling (Huang et al., 2021). A glutathione-responsive nano-prodrug has been found to simultaneously block glycolysis in tumor cells, relieving the immunosuppressive microenvironment through synergetic effects (Liu et al., 2021). In summary, glycolysis may be critical for the prognosis and treatment of COAD patients. As a signaling molecule, glycolysis-generated lactate can be released into the extracellular environment, which is closely related to tumor metastasis, poor prognosis and recurrence (Perez-Tomas and Perez-Guillen, 2020; Liao et al., 2021). Therefore, identifying highly sensitive and specific biomarkers related to glycolysis- and lactate-related genes is a promising therapeutic strategy.

In this study, we identified four glycolysis- and lactate-related molecular subtypes and constructed and validated a glycolysis- and lactate-related gene prognostic signature comprising twelve genes. Next, we assessed the accuracy and sensitivity of the glycolysis- and lactate-related gene prognostic signature using nomogram, calibration curves, DCA, and the CIC. Additionally, based on the prognostic signature, we investigated the immunological landscape, immune cell infiltration, somatic mutations, cooccurrence and mutually exclusive mutations of differentially mutated genes, and chemotherapeutic drug sensitivity in the two risk groups. Then, small-molecule drug docking molecular targets with minimal binding energies were screened. Finally, qRT–PCR assays were carried out to further verify glycolysis- and lactate-related gene expression levels. Briefly, we aimed to identify glycolysis- and lactate-related molecular subtypes and prognostic signature using bioinformatics and statistical analysis, which are highly significant for the prognosis and individualized treatment of COAD patients.

## Materials and methods

### Acquisition and processing of data

**FIGURE 1:**
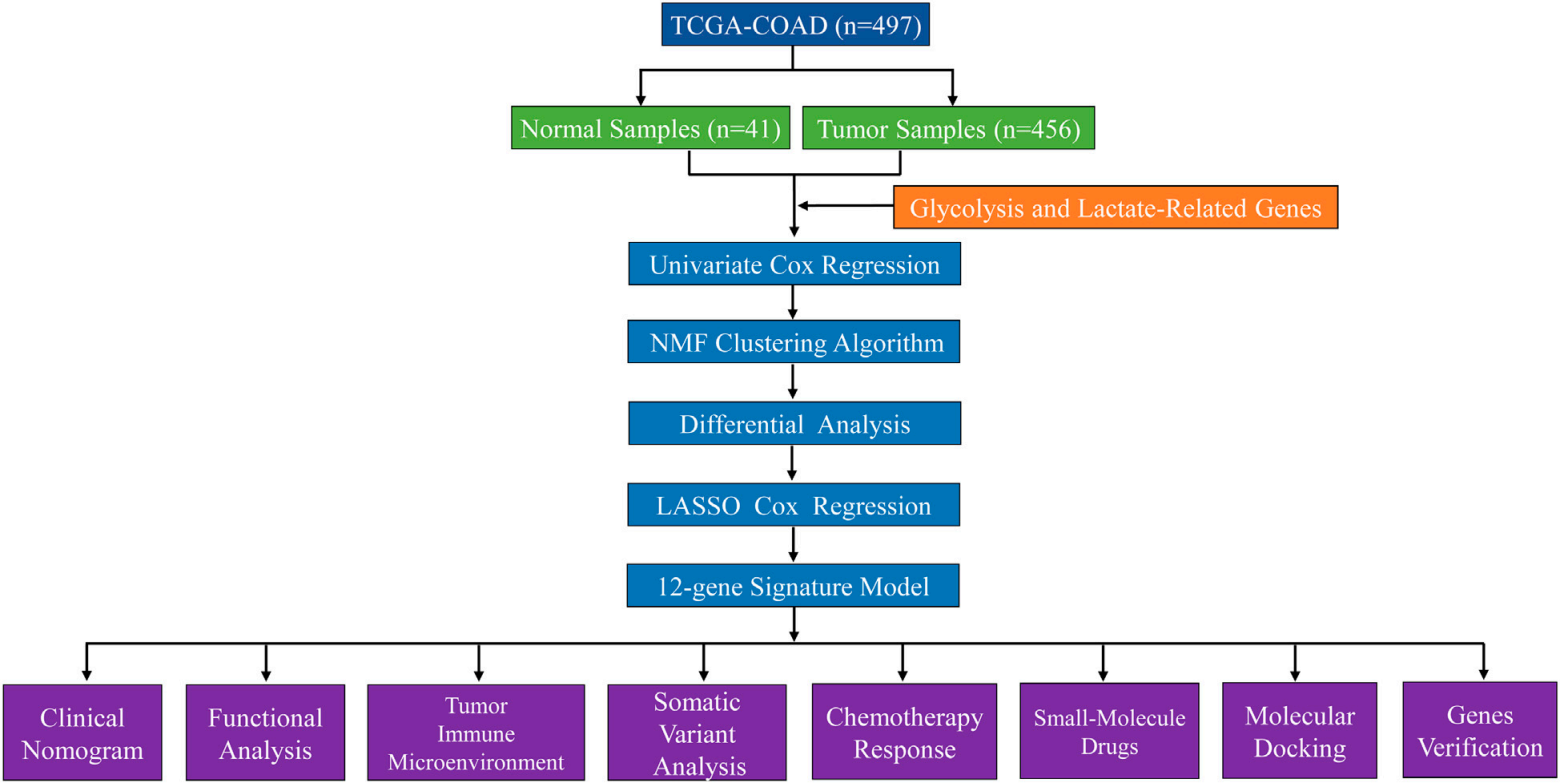
Study flow chart.

We obtained RNA sequencing transcriptome data, mutation profiles, and related clinical information for COAD patients from the TCGA database. Supplementary Table S1 provides an overview of the comprehensive clinical information of COAD patients. Patients with incomplete survival information were excluded, leaving 452 COAD patients for inclusion. Supplementary Table S2 contains a list of 458 glycolysis- and lactate-related genes extracted from the GSEA database. Thirty-nine genes related to OS were identified by univariate Cox regression analysis. Supplementary Table S3 lists thirty-nine OS-related genes, and their expression data are provided in Supplementary Table S4. The data processing and analysis flowchart is presented in Figure 1.

### Identification of molecular subtypes using the nonnegative matrix factorization algorithm

COAD patients were clustered using the nonnegative matrix factorization (NMF) clustering algorithm. The default “brunet” option was chosen, and 50 iterations were executed. The number of clusters (K) varied from 2 to 10, the average contour of the common membership matrix was calculated using the “NMF” R package, and the minimum number of subclass members was set at 10. Using cophenetic, scatter, and silhouette characteristics, *n* = 4 was determined as the optimal clustering number.

### Analysis of differentially expressed glycolysis- and lactate-related genes

Based on the “limma” R package, the differentially expressed genes (DEGs) were identified among the best prognostic C2 and C1/C3 molecular subtypes (|log_2_ FC| > 1 and FDR < 0.05). Twenty-four genes were identified in the intersection of the DEGs (Supplementary Table S5).

### Establishment and validation of a glycolysis- and lactate-related gene prognostic signature

Furthermore, twenty-four differentially expressed glycolysis- and lactate-related genes were subjected to least absolute shrinkage and selection operator (LASSO) Cox regression analysis using the R package “glmnet”. We established a glycolysis- and lactate-related gene prognostic signature comprising twelve critical genes. Next, we calculated the risk score for each COAD patient. High- and low-risk COAD patients were grouped by the median risk score. We evaluated the prognostic performance of glycolysis- and lactate-related gene prognostic signature using Kaplan–Meier (KM) analysis and compared them in the high- and low-risk groups. Using the “timeROC” R package, we plotted time-dependent receiver operating characteristic (ROC) curves. Next, the STRING database (https://www.string-db.org/) was used to construct a protein–protein interaction (PPI) network, which was visualized by using Cytoscape software (Version 3.7.1).

### Construction and evaluation of clinical nomogram

A prognostic nomogram was constructed by integrating the risk score, age, gender, and stage using the R packages “rms” and “regplot”. The nomogram was used to predict the 1-, 3-, and 5-year survival rates of COAD patients. We plotted the calibration curves to evaluate the predictive accuracy of the nomogram. We evaluated the clinical relevance of the prognostic signature using decision curve analysis (DCA) and a clinical impact curve (CIC).

### Immune landscape analysis

The proportions of twenty-two immune cell subpopulations in COAD cohorts were calculated using the CIBERSORT algorithm. Additionally, we analyzed the correlation of the cytolytic (CYT) activity score with the prognostic signature. We conducted single-sample GSEA (ssGSEA) analyses for each sample to determine its level of immune cell activity. MHC molecules and immune checkpoints (ICPs) were compared between the high- and low-risk groups.

### Anticancer immune response analysis

The anticancer immune response in the tumor microenvironment involves seven steps. We calculated the activity scores for each step using the Tracking Tumor Immunophenotype database (TIP; http://biocc.hrbmu.edu.cn/TIP/). Next, we assessed anticancer immune responses by comparing the activity scores of the seven steps between the high- and low-risk groups.

### Somatic variant analysis

We used the VarScan platform data from the TCGA-COAD cohort to analyze the somatic mutation data for each patient. Next, the “maftools” package was used to visualize the mutations in the two risk groups. Additionally, a correlation analysis of the tumor mutational burden (TMB) and the prognostic signature was performed.

### Analysis of chemotherapy drug susceptibility and identification of small-molecule drugs

The Genomics of Drug Sensitivity in Cancer (GDSC; https://www.cancerrxgene.org/) database was used to predict the chemotherapy response for COAD patients. To assess the therapeutic effects of chemotherapy drugs in COAD patients, the half maximal inhibitory concentration (IC50) was calculated using ridge regression with the GDSC database and the R package “pRRophetic”. Based on the DEGs in the high- and low-risk groups, we uploaded these DEGs to the Connectivity Map (cMap) database to screen small-molecule agents that might reverse tumor biological progression. *p* < 0.05 and enrichment scores ranging from −1 to 0 indicated potential drugs for COAD treatment. The structures of small-molecule drugs were obtained from the PubChem database.

### Molecular docking verification

Based on the top ten most significant DEGs, we uploaded the DEGs to the cMAP database and selected the identifiable genes. Next, we screened experimentally validated proteins from the Unified Protein Database (UniProt, https://www.uniprot.org/), downloaded protein structures from the Protein Data Bank (PDB, http://www.rcsb.org/pdb) database, and finally obtained four core molecular targets—DEFA5, CEACAM7, CLCA1, and ZG16 (Supplementary Table S6). We used Chem3D software (Version 15.0; Cambridge, MA, United States) to calculate the protein structure with the lowest binding energy. AutoDock Tools (ADT) version 1.57 software was used for processing and molecular docking. The molecular docking results were visualized using PyMOL software (https://pymol.org/2/).

### Tissue samples and quantitative real-time polymerase chain reaction (qRT–PCR)

Our study was approved by The First Affiliated Hospital of Nanchang University Ethics Committee on Medical Research. We collected COAD specimens and adjacent normal specimens from the operating room of The First Affiliated Hospital of Nanchang University. Each patient signed an informed consent form. qRT–PCR was used to analyze the quantitative expression of key prognostic genes. Total RNA was extracted with TRIzol reagent (Invitrogen, Carlsbad, CA, United States). The FastKing RT Kit (TIANGEN BIOTECH BEIJING CO., Ltd.) was used for the reverse transcription reaction. Hieff UNICON Universal Blue qPCR SYBR Green Master Mix (Yeasen Biotechnology Shanghai Co., Ltd.) was used for the qPCR. PCR was performed using an Applied Biosystems QuantStudio 5 cycler (Thermo Fisher Scientific). We calculated the relative expression levels of genes using the 2^−△△Ct^ method. β-actin was the internal reference. The sequences of the primers (Sangon Biotech Shanghai Co., Ltd.) were as follows: ALDOB forward 5′-GTG​GAA​AAC​ACT​GAA​GAG​AAC​C-3′ and reverse 5′-TCC​TTG​GTC​TAA​CTT​GAT​TCC​C-3′; APOBEC1 forward 5′-TGT​CTG​CTC​TAC​GAA​ATC​AAG​T-3′ and reverse 5′-CGT​AGA​TCA​CTA​GAG​TCA​CAC​C-3′; CLCA1 forward 5′-CTA​AGG​ATG​ACG​GTG​TCT​ACT​C-3′ and reverse 5′-GAT​GTT​CTG​CTG​AAA​CAC​ACT​T-3′; and OLFM4 forward 5′-CTT​GGT​AGA​GAA​GCT​TGA​GAC​A-3′ and reverse 5′-GGT​GTT​TTG​ATC​TTT​AGA​GGC​C-3′.

### Statistical analysis

Statistical analysis and graph visualization were carried out using R software. To determine related genes and their prognostic value, univariate Cox regression analysis was performed. Kaplan–Meier survival analysis was performed for OS. We compared the results between groups using log-rank tests. Based on Spearman’s correlation analysis, the association between the prognostic signature and immune score was identified. A value of *p <* 0.05 was considered significant.

## Results

### Identification of glycolysis- and lactate-related molecular subtypes

**FIGURE 2:**
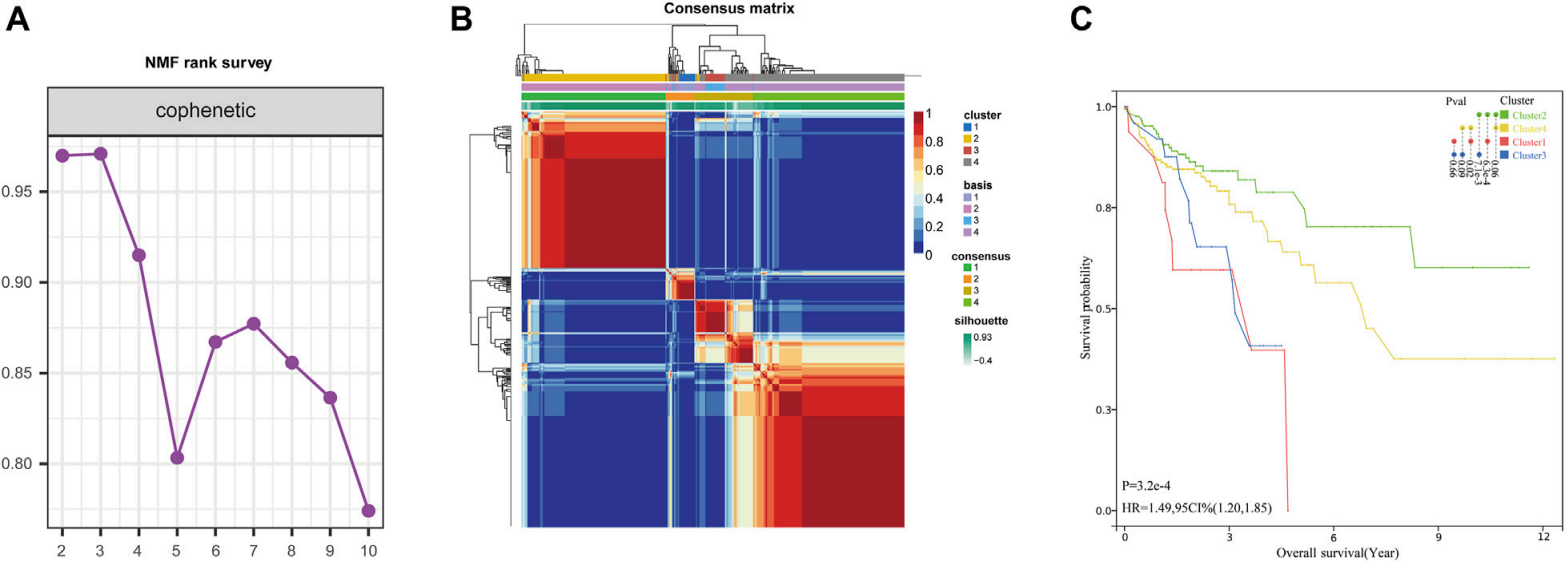
Identification of glycolysis- and lactate-related molecular subtypes. **(A)** Consensus map of NMF clustering. **(B)** Different glycolysis- and lactate-related molecular subtypes of the TCGA cohort were identified for k = 4. **(C)** Survival analyses of the four clusters.

Based on the 458 glycolysis- and lactate-related genes downloaded from the GSEA database, clinical data from 452 COAD patients were obtained from the TCGA database. Using univariate Cox regression analysis, we first identified thirty-nine survival-related genes. Next, based on the NMF algorithm, four molecular subtypes of COAD were identified. Using the indicator of basis, consensus, and silhouette, four clusters were identified as optimal. The intergroup correlations were low, and the intragroup correlations were the highest (Figures 2A,B). We further analyzed the prognosis associated with the four molecular subtypes. KM survival analyses showed that cluster two had the best prognosis, and clusters one and three had poor prognoses (Figure 2C).

### Differential expression and analysis of glycolysis- and lactate-related genes among the four clusters

**FIGURE 3:**
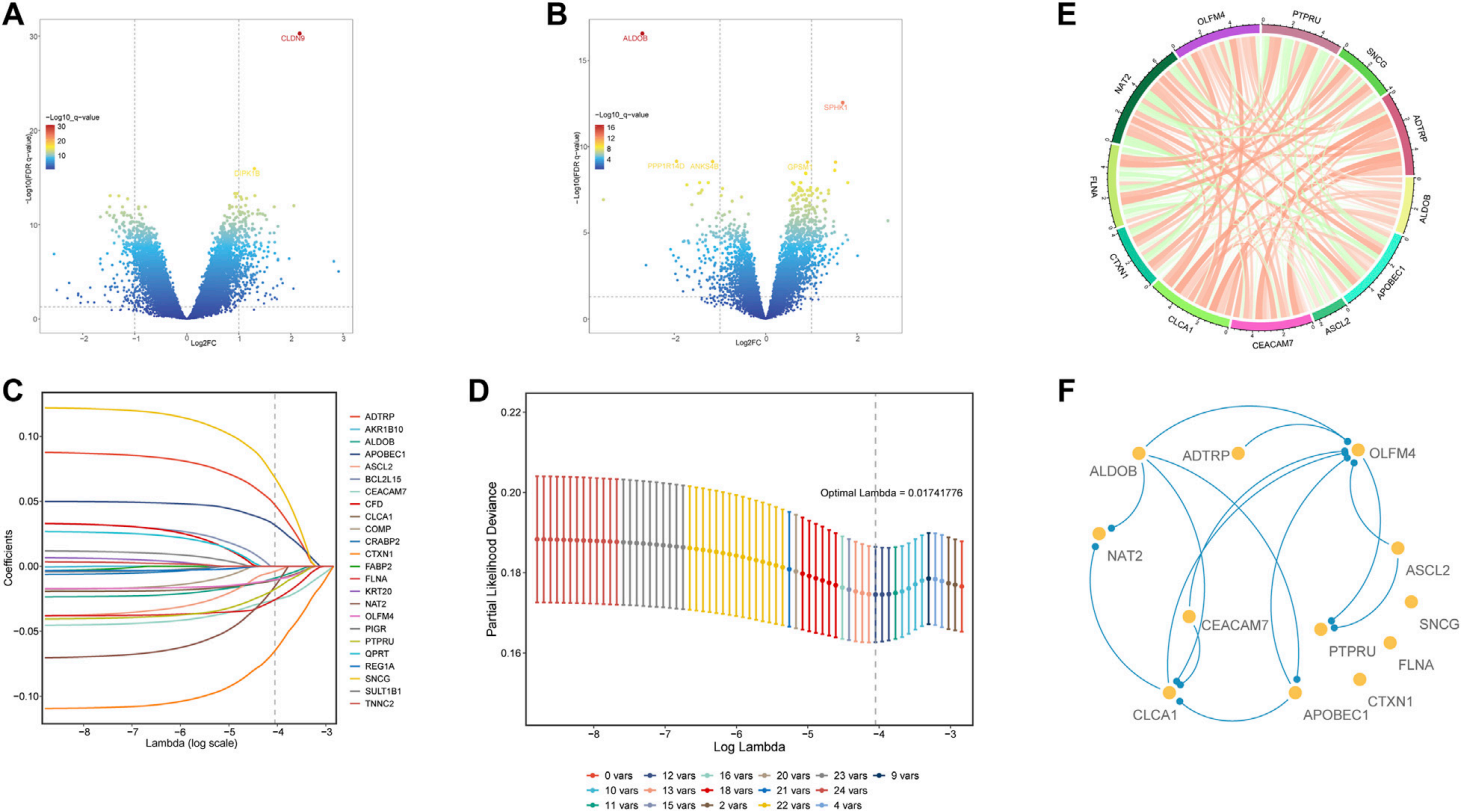
Differential expression and analysis of glycolysis- and lactate-related genes among the four clusters. **(A)** DEGs between clusters 1 and 2. **(B)** DEGs between clusters 3 and 2. **(C)** LASSO coefficient profile of twelve OS-related glycolysis- and lactate-related genes. **(D)** Optimal tuning parameter of glycolysis- and lactate-related genes. **(E)** Gene expression correlations of twelve OS-related glycolysis- and lactate-related genes. **(F)** PPI network analysis of twelve OS-related glycolysis- and lactate-related genes.

To further screen for differential glycolysis- and lactate-related genes, we performed differential gene expression analysis between cluster two and cluster one or cluster three and identified the intersecting DEGs to obtain twenty-four genes associated with a poor prognosis (Figures 3A,B). Next, using LASSO Cox regression, twelve glycolysis- and lactate-related genes were screened based on 1,000-fold tenfold cross-validation to construct the prognostic signature (Figures 3C,D). Additionally, Spearman’s correlation analysis was applied to evaluate the expression correlation of twelve glycolysis- and lactate-related genes. ADTRP and CLCA1 exhibited a positive correlation, and PTPRU and SNCG exhibited a negative correlation (Figure 3E)**.** Finally, PPI analysis was conducted to explore the interactions among the twelve glycolysis- and lactate-related DEGs (Figure 3F). More lines between genes indicate that more interactions exist with other genes. We identified OLFM4 and CLCA1 as hub genes (Figure 3F).

### Development and validation of a glycolysis- and lactate-related gene prognostic signature for colon adenocarcinoma

**FIGURE 4:**
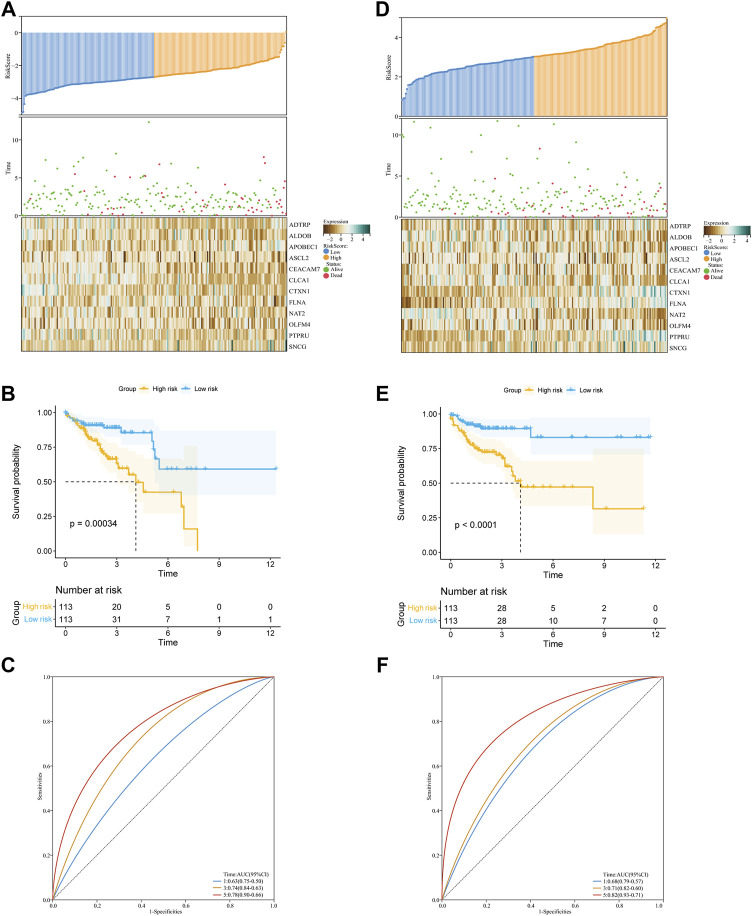
Construction and validation of the glycolysis- and lactate-related gene prognostic signature. **(A,D)** Predictive ability of the prognostic signature in the training cohort **(A)** and validation cohort **(D)**. Distribution of the risk score (upper), survival time (middle), and heatmap of selected glycolysis- and lactate-related genes (below). **(B,E)** KM survival curves of OS for COAD patients between the training cohort **(B)** and validation cohort **(E). (C,F)** ROC curves for the 1-, 3-, and 5-year survival in the training cohort **(C)** and validation cohort **(F)**.

First, we calculated the risk score for each COAD patient using the following formula: Risk score = (0.248063077) * ADTRP + (−0.133258741) * ALDOB + (0.220438097) * APOBEC1 + (−0.01464853) * ASCL2 + (−0.000764405) * CEACAM7 + (−0.084507044) * CLCA1 + (−0.033058311) * CTXN1 + (0.078872672) * FLNA + (−0.289695621) * NAT2 + (0.002887704) * OLFM4 + (0.046736691) * PTPRU + (0.126164132) * SNCG. Then, we constructed a glycolysis- and lactate-related gene prognostic signature comprising twelve glycolysis- and lactate-related genes in the training cohort. Based on their median risk score, COAD patients were categorized into high-risk and low-risk groups. Figure 4A illustrates the distribution of risk scores and survival status of each patient. We used a heatmap to show the differential expression of the twelve glycolysis- and lactate-related genes (Figure 4A). According to the KM survival curves, high-risk patients showed a lower OS than low-risk patients (Figure 4B). In the training cohort, the areas under the ROC curves (AUCs) for the 1-, 3-, and 5-year OS were 0.63, 0.74, and 0.78, respectively, indicating that the signature may serve as a prognostic indicator for COAD patients (Figure 4C).

Similarly, we performed analyses in the validation cohort and entire cohort. Figure 4D depicts the risk score distribution, survival status, and glycolysis- and lactate-related gene expression profiles in the validation cohort and entire cohort. Our results revealed that the low-risk group had better OS than the high-risk group (Figure 4E). For the two cohorts, the AUCs for the 1-, 3-, and 5-year OS were 0.68, 0.71, and 0.82 and 0.66, 0.63, and 0.66, respectively (Figure 4F). Furthermore, stratification analyses based on age, gender, and stage showed that the OS of low-risk patients was better than that of high-risk patients (Supplementary Figure S1). To further evaluate the predictive ability of the glycolysis- and lactate-related gene prognostic signature, we applied two independent external datasets (GSE17536 and GSE39582) to validate our findings. As shown in Supplementary Figures S2A, B, the KM curve analyses indicated that the OS of high-risk patients was significantly worse than that of low-risk patients, which was consistent with our analysis in the TCGA-COAD cohort.

### Correlation between the glycolysis- and lactate-related gene prognostic signature and clinical characteristics

**FIGURE 5:**
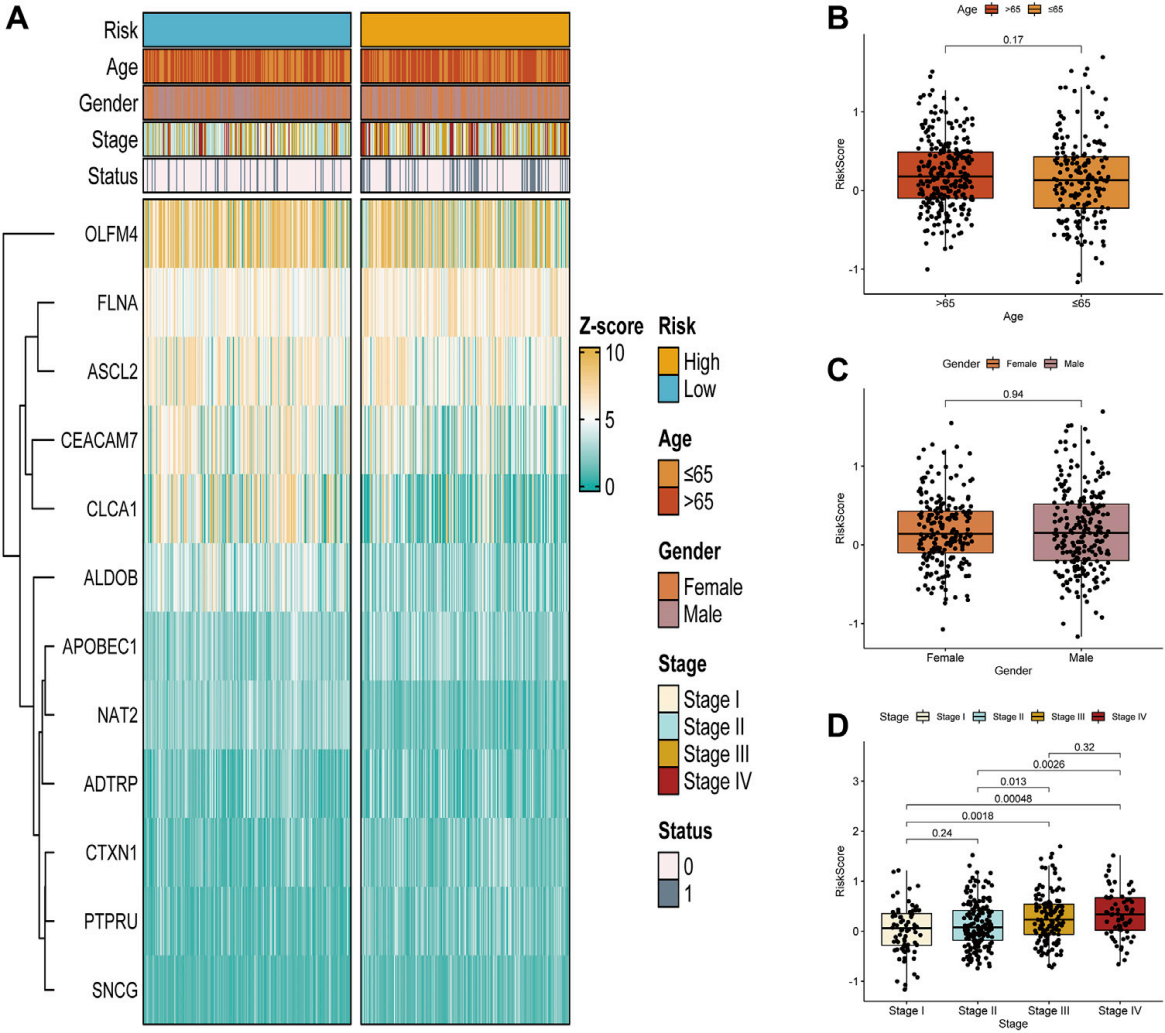
Correlation between the glycolysis- and lactate-related gene prognostic signature and clinical characteristics. **(A)** Differences in glycolysis- and lactate-related gene expression and clinical characteristics between the two risk groups. **(B–D)** Analysis of correlations between the glycolysis- and lactate-related gene signature and age **(B)**, gender **(C)**, and stage **(D)**.

Next, Figure 5A demonstrates the association of clinical characteristics with prognostic gene expression levels between the high- and low-risk groups. It was evident that the two risk groups had large differences. We further explored the value of the prognostic signature in the two risk groups stratified by different clinical characteristics (age, gender, and stage). Age and the risk score did not differ significantly (Figure 5B). Additionally, the risk score and gender were not significantly correlated (Figure 5C). Furthermore, significant correlations were found between stage I and stage III and the risk score. The risk scores of stage I and stage IV were significantly different, the risk scores of stage II and stage III were significantly different, and the risk scores of stage II and stage IV were significantly different (Figure 5D). In summary, the risk scores were significantly different for different stages.

### Clinical nomogram for predicting the survival times of colon adenocarcinoma patients

**FIGURE 6:**
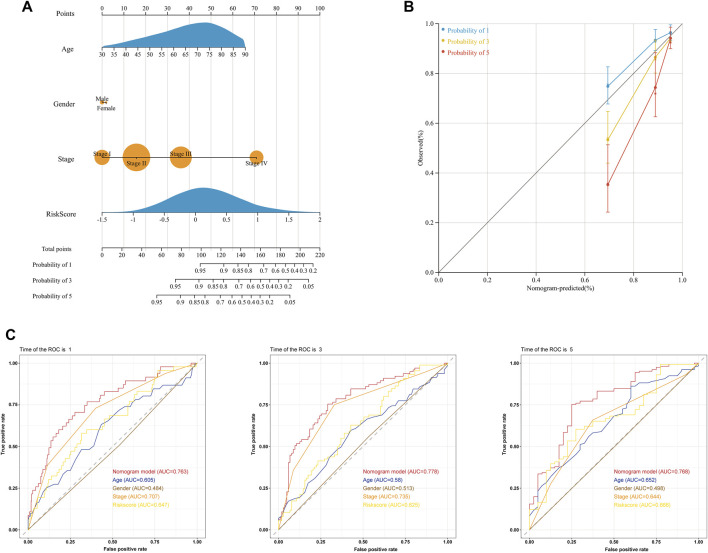
Prediction of the OS of COAD patients using a clinical nomogram. **(A)** Nomogram for predicting the 1-, 3-, and 5-year OS of COAD patients. **(B)** Calibration curves of the nomogram for predicting OS at 1, 3, and 5 years. Nomogram-predicted survival is on the X-axis, and actual survival is on the Y-axis. **(C)** ROC curves of the nomogram for predicting the 1-, 3- and 5-year OS.

We conducted univariate and multivariate Cox regression analyses to investigate whether the risk score was an independent predictor of OS for COAD patients. The results of univariate Cox regression indicated that the risk score was significantly related to OS (Supplementary Figure S3A). Additionally, using multivariate Cox regression, the risk score independently predicted the OS of COAD patients (Supplementary Figure S3B). Subsequently, we developed a clinical nomogram to determine the 1-, 3-, and 5-year OS of COAD patients on account of a combination of clinicopathological features and glycolysis and the lactate-related gene prognostic signature (Figure 6A). Calibration curves showed excellent agreement between actual observations and the predicted rates of the 1-, 3-, and 5-year OS (Figure 6B). Regarding the nomogram, the AUCs of the 1-, 3- and 5-year OS were 0.763, 0.778, and 0.768, respectively (Figure 6C). The DCA curve revealed that the nomogram was superior to other clinical characteristics (Supplementary Figure S3C). Additionally, the CIC visually demonstrated that the nomogram had a high clinical net benefit, confirming that the nomogram can accurately predict patient prognosis and can be used to guide clinical decision-making (Supplementary Figure S3D).

### Functional analysis of the signature

**FIGURE 7:**
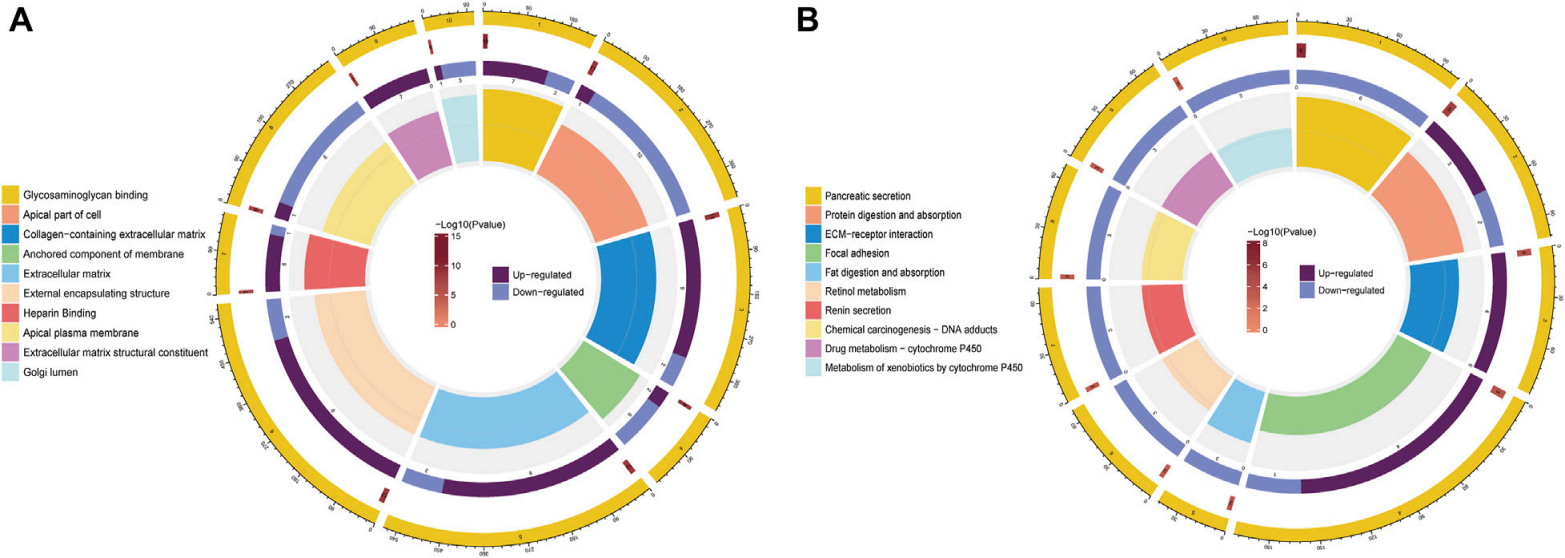
Functional enrichment analysis of DEGs between the high- and low-risk groups. **(A)** Analysis of DEGs for GO enrichment. **(B)** Analysis of KEGG enrichment for DEGs.

GO and KEGG pathway enrichment analyses of DEGs were conducted in the high- and low-risk groups. GO terms were mainly enriched in “glycosaminoglycan binding,” “apical part of cell,” “external encapsulating structure,” “apical plasma membrane,” and “collagen-containing extracellular matrix” (Figure 7A). Additionally, KEGG pathway enrichment showed that DEGs were primarily involved in “pancreatic secretion,” “protein digestion and absorption,” “focal adhesion,” “renin secretion,” and “drug metabolism—cytochrome P450” (Figure 7B).

### Interrelationship between the glycolysis- and lactate-related gene prognostic signature and immune cell infiltration

**FIGURE 8:**
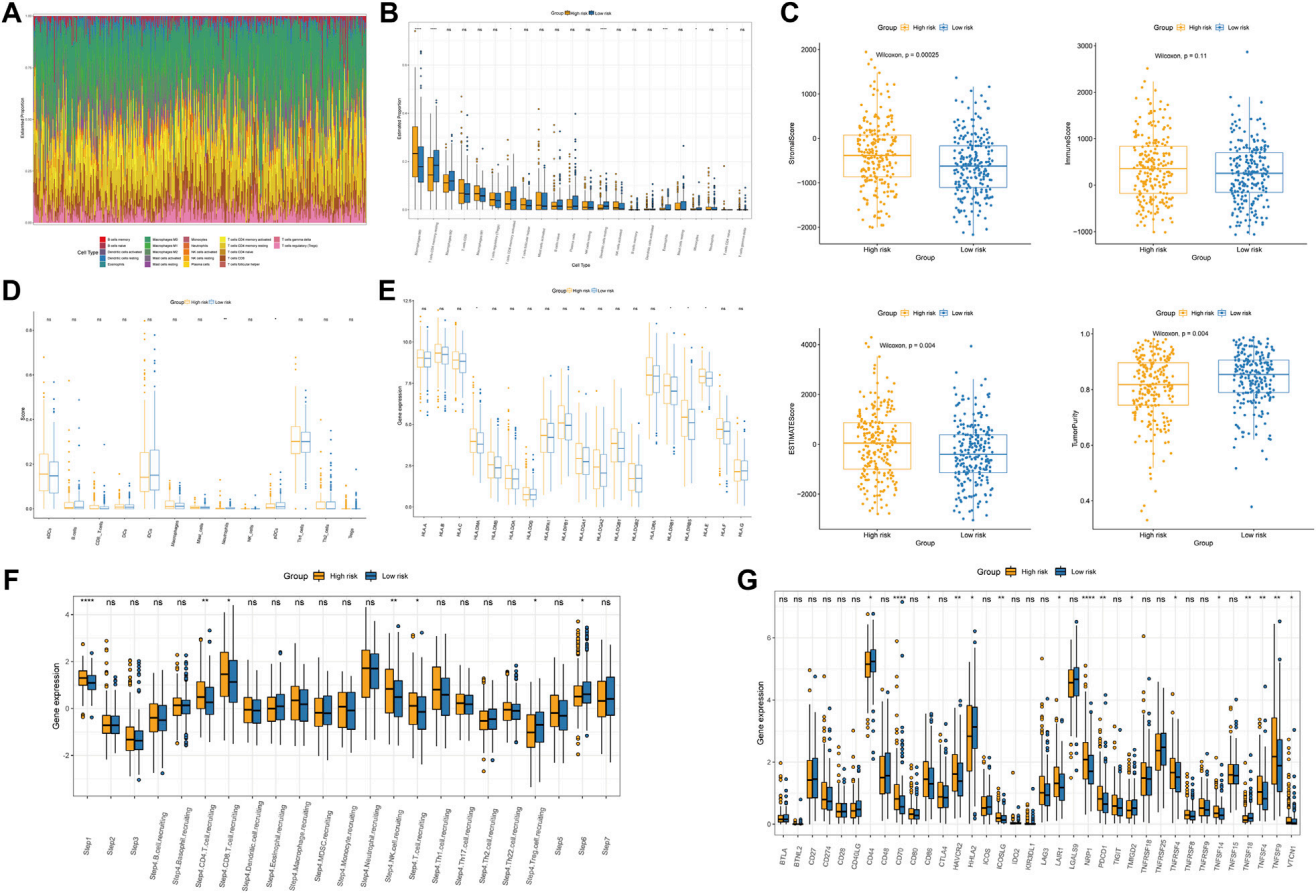
Immune profiles between the two subgroups. **(A)** Immune cell proportions in COAD patients. **(B)** Analysis of twenty-two types of tumor-infiltrating immune cells. **(C)** Boxplot of the differences in the stromal score, immune score, ESTIMATE score, and tumor purity. **(D)** Comparison of the levels of infiltration of immune cells in the two groups. **(E)** Differences in MHC molecule expression. **(F)** Relative abundance of the antitumor immune response between the high- and low-risk groups. **(G)** Immune checkpoint expression in the high- and low-risk groups. *p < 0.05; **p < 0.01; ***p < 0.001; ****p < 0.0001; ns, not significant.

Using the CIBERSORT algorithm, we comprehensively assessed the relationship between the risk score and twenty-two types of immune cells. Figure 8A depicts the immune landscape of the dominant types of immune cells in COAD. The visualization results showed that macrophages, resting CD4+ T cells, and activated CD4+ T cells constituted most of the immune cells. CD4 memory resting T cells, CD4 memory activated T cells, and resting dendritic cells were significantly higher in the low-risk group than the high-risk group (Figure 8B). A higher percentage of M0 macrophages was observed in the high-risk group than in the low-risk group. Compared with the low-risk group, the high-risk group had higher stromal, immune, and ESTIMATE scores but a lower tumor purity score (Figure 8C). We also compared the distribution of the CYT activity score between the two risk groups. The CYT activity score of the high-risk group was higher than that of the low-risk group (Supplementary Figure S4A). Using ssGSEA algorithms, we analyzed the RNA sequencing data from COAD patients for evidence of immune cell infiltration. Neutrophils and plasmacytoid dendritic cells (pDCs) were significantly different between the two groups (Figure 8D). Additionally, the low-risk group had significantly lower amounts of MHC molecules (Figure 8E). To assess the impact of immune cells on CAOD prognosis, we analyzed the expression of antitumor immune cells between the two risk groups. The antitumor immune cells were significantly different between the two groups and were mainly higher in the high-risk group than in the low-risk group (Figure 8F). Considering that the TMB is closely related to cancer immunotherapy, we calculated the TMB values of each patient between the two risk groups based on the prognostic signature. As expected, the TMB quantification analysis showed that the high-risk group possessed a higher TMB (Supplementary Figure S4B). Given the importance of ICPs in immunotherapy, we further evaluated the expression differences of ICPs. As shown in Figure 8G, there were significant differences in CD70, HAVCR2, LAIR1 and NRP1 between the high- and low-risk groups.

### Differentiation of mutated genes between the two risk subgroups

**FIGURE 9:**
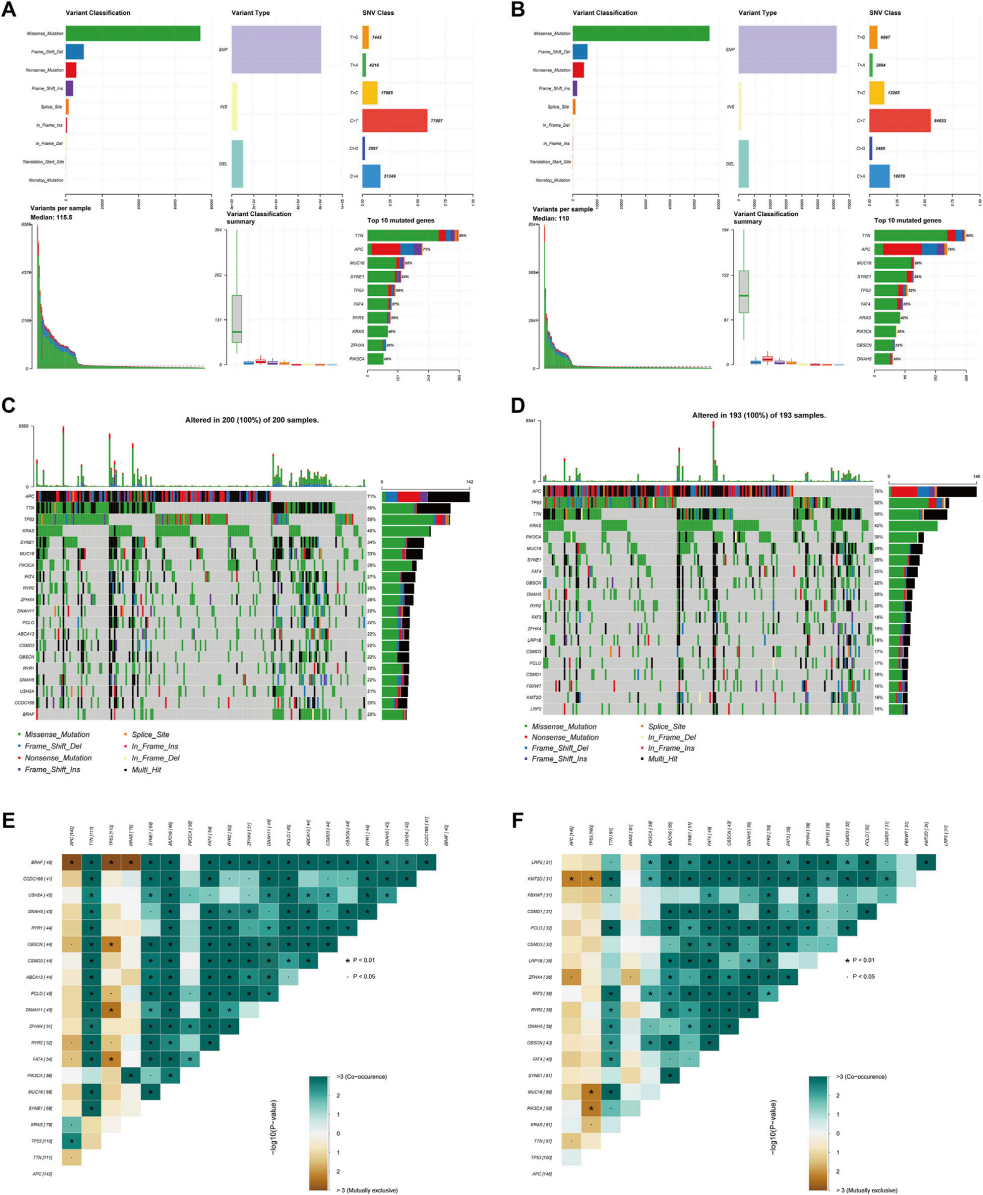
Somatic mutation analysis in the two subgroups. **(A,B)** Distribution of mutation types between the high-risk **(A)** and low-risk groups **(B)**. The upper panel depicts the variant classification, variant type, and SNV class of mutated genes, and the bottom panel represents variants per sample, variant classification, and the top ten mutated genes. **(C,D)** Waterfall plot of somatic mutations between the high-risk **(C)** and low-risk groups **(D)**. **(E,F)** Comparison of co-occurrence and mutually exclusive mutations of the mutated genes between the high-risk **(E)** and low-risk groups **(F)**.

Figures 9A,B summarizes the information regarding mutated genes according to the variant classification, variant type, and single nucleotide variant (SNV) class. The waterfall plot shows the mutational landscape of the top twenty genes in the high- and low-risk groups with the highest mutation frequencies. The most frequent mutations were found in APC, followed by TTN, TP53, and KRAS in the high-risk group (Figure 9C). All 193 samples (100%) of the low-risk group had increased expression of APC (76%) and TP53 (52%) as well as TTN (50%) over other altered genes (Figure 9D). Additionally, we detected somatic mutation interactions. The high-risk group had several gene mutations, including mutually exclusive APC-BRAF mutations (Figure 9E). We also identified mutually exclusive TP53-MUC16 mutations in the low-risk group (Figure 9F).

### Chemotherapeutic drug sensitivity analysis and small-molecule drug screening

**FIGURE 10:**
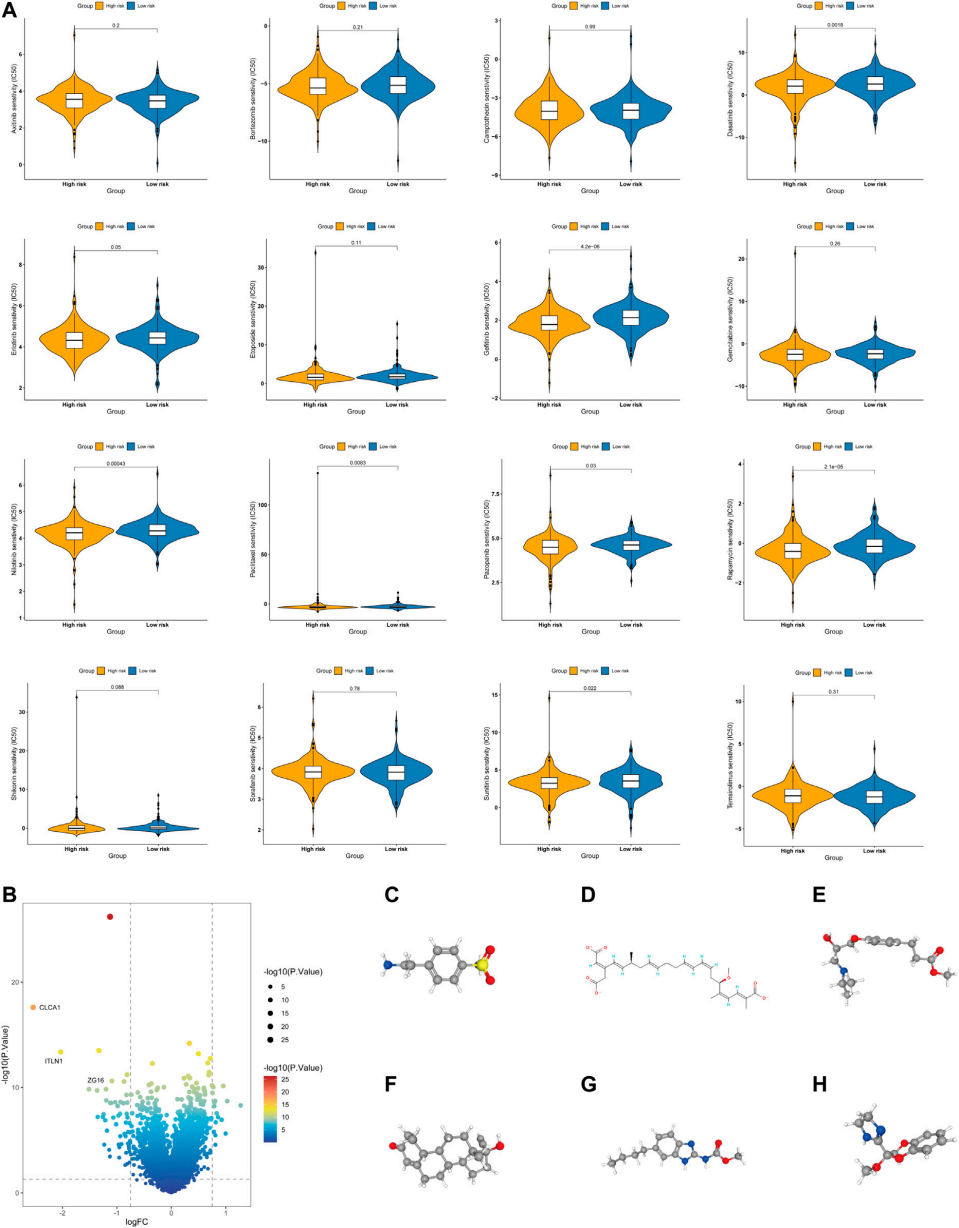
Evaluation of the chemotherapy response and screening of small-molecule drugs. **(A)** Sensitivity analysis of chemotherapy drugs between the high- and low-risk groups. **(B)** Volcano plot of DEGs between the high- and low-risk groups. **(C–H)** Structures of six small-molecule drug candidates, namely, 4-(2-aminoethyl) benzenesulfonamide **(C)**, bongkrek acid **(D)**, esmolol **(E)**, norethisterone **(F)**, parbendazole **(G)**, and RX-821002 **(H)**.

Chemotherapy drugs have remained the primary treatment for COAD. A leading cause of the poor prognosis among COAD patients is chemoresistance. In this study, we investigated the sensitivities of patients in both the high- and low-risk groups to common chemotherapy drugs for treating COAD. Interestingly, the low-risk group exhibited increased IC50 values for paclitaxel, dasatinib, gefitinib, nilotinib, pazopanib, rapamycin, and sunitinib, indicating that high-risk patients may benefit from these chemotherapy agents. Overall, these results showed that the glycolysis- and lactate-related gene signature was related to drug sensitivity (Figure 10A).

Additionally, we screened potential small-molecule agents that could be used to treat COAD using the cMAP database. The volcano plot shows the DEGs in the high- and low-risk groups, including sixteen upregulated genes and fifty-five downregulated genes (Figure 10B). Based on these DEGs, we screened six potential small-molecule drugs, namely, 4-(2-aminoethyl) benzenesulfonamide, bongkrek acid, esmolol, norethisterone, parbendazole, and RX-821002. Figures 10C–H displays the structures of the six small-molecule drugs based on the PubChem database.

### Molecular docking verification

**FIGURE 11:**
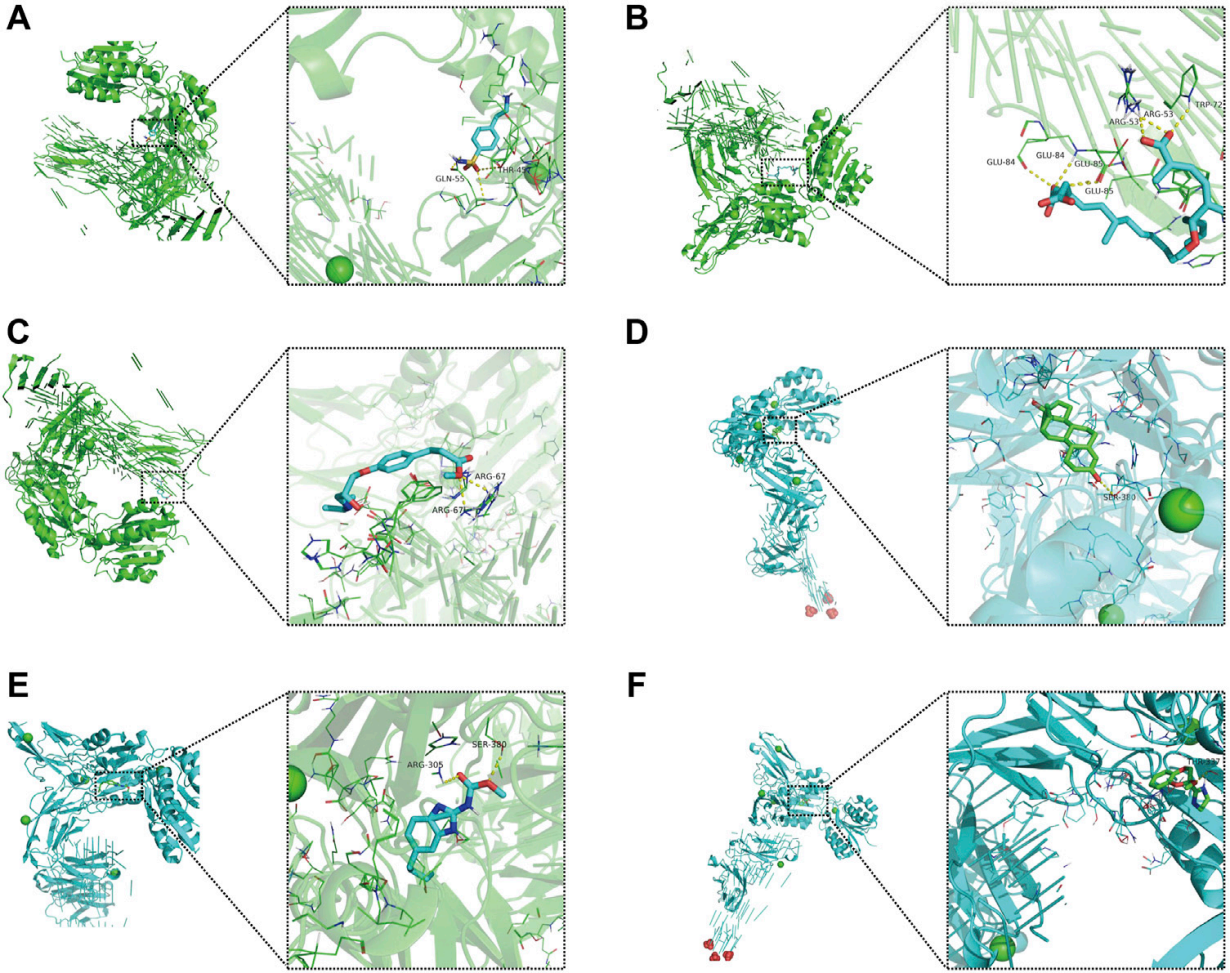
Molecular docking of small-molecule drugs and core molecular targets. **(A)** Parbendazole-CLCA1. **(B)** 4-(2-Aminoethyl) benzenesulfonamide-ZG16. **(C)** Bongkrek-acid-ZG16. **(D)** Norethisterone-CLCA1. **(E)** Esmolol-ZG16. **(F)** RX-821002-CLCA1.

Molecular docking is an in silico structure-based computational method for drug screening. Using AutoDock Tools1.57 software, we docked four core molecular targets (DEFA5, CEACAM7, CLCA1, and ZG16) and screened six small-molecule drugs. Studies have shown that the lower the binding energy of receptors and ligands is, the more stable the binding conformation and the higher the possibility of action. Therefore, we used PyMOL software to visualize small-molecule drug docking molecular targets with minimal binding energies (Figures 11A–F). For example, bongkrek acid may bind to ZG16 and form hydrogen bonds with amino acid residues GLU-84, GLU-85, ARG-53 and TRP-72 near the active site to exert its biological functions.

### Expression of prognostic genes

**FIGURE 12:**
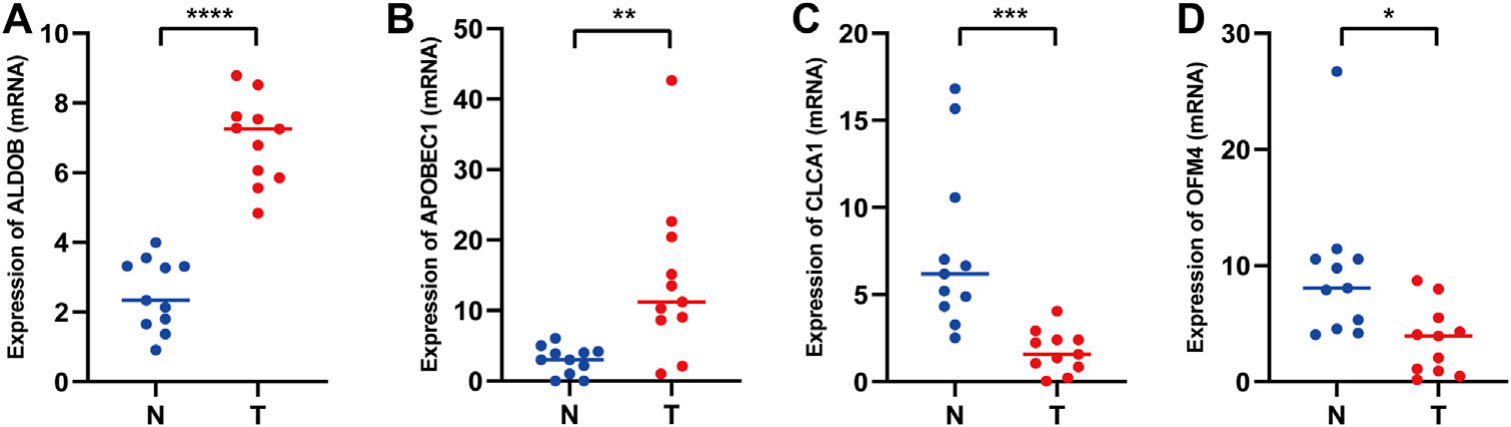
mRNA expression levels of glycolysis- and lactate-related prognostic genes in COAD tissues and adjacent normal tissues. **(A–D)** Relative mRNA expression levels of ALDOB **(A)**, APOBEC1 **(B)**, CLCA1 **(C)**, and OLFM4 **(D)** in COAD tissues and adjacent normal tissues. T, COAD tissues; N, adjacent normal tissues. *p < 0.05; **p < 0.01; ***p < 0.001; ****p < 0.0001.

To further verify the important roles of glycolysis- and lactate-related genes in COAD, we examined the expression of related key genes in 11 pairs of COAD specimens and adjacent normal specimens. As shown in Figures 12A,B, we found that ALDOB and APOBEC1 were elevated in COAD samples. However, CLCA1 and OLFM4 had increased expression in normal samples (Figures 12C,D). In addition, we obtained the protein expression of glycolysis- and lactate-related genes from the Human Protein Atlas database. As shown in Supplementary Figure S5, representative immunohistochemistry images showed that PTPRU and ALDOB were highly expressed in COAD tissues, while CLCA1, CTXN1, FLNA, SNCG, NAT2, ADTRP, CEACAM7, and OLFM4 were highly expressed in normal tissues.

## Discussion

COAD patients show high morbidity and mortality rates because of metastasis, cancer recurrence, and chemotherapy resistance (Hammond et al., 2016; Snyder et al., 2021). An increasing number of studies have suggested that a typical characteristic of malignancy is abnormal metabolism, which can confer growth advantages to tumor cells by remodeling the tumor microenvironment (Martinez-Reyes and Chandel, 2021). The Warburg effect indicates that unlike normal differentiated cells, tumor cells also rely on glycolysis for energy, even under conditions of sufficient oxygen (Pascale et al., 2020). This aberrant glycolysis in tumor cells can promote glucose uptake and lactate production so that tumor cells obtain energy for metabolic and survival processes, further promoting tumor development and growth (Vaupel and Multhoff, 2021). A primary objective of this study was to investigate the role of glycolysis- and lactate-related genes in COAD patient OS, immune microenvironment, and chemotherapeutic sensitivity to drugs. Our study, which analyzed the TCGA and GSEA databases, identified four molecular subtypes and generated a glycolysis- and lactate-related gene prognostic signature. The high-risk group of patients exhibited a poor prognosis, low levels of immune cell infiltration, high levels of somatic mutations, and a high sensitivity to chemotherapeutics.

We developed a prognostic model based on 12 glycolysis- and lactate-related genes (ADTRP, ALDOB, APOBEC1, ASCL2, CEACAM7, CLCA1, CTXN1, FLNA, NAT2, OLFM4, PTPRU, and SNCG), and the combination of these genes generated an accurate prediction of the clinical outcome. By hydrolyzing fatty acid esters of hydroxy-fatty acids, ADTRP can prevent diabetes and inflammation and control metabolic diseases (Lupu et al., 2021). Colorectal cancer (CRC) cells may undergo metabolic reprogramming during liver colonization when the liver environment is conducive to aldolase B upregulation. This upregulation enhances fructose metabolism and promotes the growth of liver metastases caused by CRC (Bu et al., 2018). The expression levels of APOBEC1 can be used to predict pancancer outcomes, particularly for patients with pancreatic and thyroid carcinoma (Niavarani et al., 2018). In CRC, ASCL2 overexpression may increase the tendency to self-renew rather than differentiate, causing liver metastases to become self-renewing (Stange et al., 2010). CAR T-cell therapy targeting CEACAM7 has been reported as a potential treatment for pancreatic ductal adenocarcinomas (Raj et al., 2021). Inhibition of the Wnt/beta-catenin signaling pathway and epithelial-mesenchymal transition by CLCA1 can reduce CRC aggressiveness (Li et al., 2017). In a chronic ischemia model, CTXN reduced the size of necrosis of brain tissue, improved the functioning of the antioxidant system and reduced neurodegenerative changes (Kurkin et al., 2021). In CRC, FLNA induces epithelial-mesenchymal transition, which activates the smad2 pathway, increasing chemoresistance (Cheng et al., 2020). CRC metastasis is closely related to the expression level of the NAT2 gene, which can be used as a biomarker for prognosis and therapy (Wang et al., 2021). Early gastric cancers with high OLFM4 expression may have an increased risk of metastasis to the lymph nodes, and combining OLFM4 expression with tumor size and differentiation status may result in better classification of early gastric cancer patients (Zhao et al., 2016). By inhibiting Hippo/YAP signaling, PTPRU functions as a tumor suppressant that inhibits the stemness of cancer stem cells (Gu et al., 2019). In high-grade serous ovarian cancer, SNCG upregulation contributes to poor clinical outcomes. These findings demonstrated that SNCG plays a pivotal role in promoting metastasis by activating the PI3K/Akt signaling pathway (Zhang et al., 2020). Therefore, glycolysis- and lactate-related genes are involved in the development of COAD and are potentially useful as markers in clinical settings.

Further analysis revealed that the glycolysis- and lactate-related gene prognostic signature was robust and accurate. Patient survival was longer for patients in the low-risk group than for those in the high-risk group in our study. Based on the 3-year OS, the AUCs of the training and validation cohorts were 0.74 and 0.71, respectively, suggesting that our model is superior to those in other studies (AUC = 0.676, AUC = 0.63) (Qiu et al., 2021; Xia et al., 2021). Therefore, our prognostic signature has excellent predictive power and accuracy compared with other prognostic signatures developed by other investigators. Additionally, COAD patients’ 1-, 3-, and 5-year OS can be accurately predicted using a nomogram that incorporates risk scores and clinical characteristics. The AUC values revealed that the nomogram is superior to other independent factors that can assist clinicians in making decisions and devising personalized treatment strategies for COAD patients.

COAD recurrence and metastasis are influenced by the tumor microenvironment, particularly the immune microenvironment (Xiong et al., 2019; Mei et al., 2021). Increasing research has highlighted the role of immune cell infiltration in the development, metastasis, and immunosuppression of COAD (Picard et al., 2020). The current study investigated the correlation between tumor-infiltrating cells and glycolysis- and lactate-related gene prognostic signatures. Utilizing the CIBERSORT algorithm, a comprehensive evaluation of the abundance and infiltration of twenty-two immune cells was conducted in COAD patients. The low-risk group exhibited a higher proportion of dendritic cells and CD4+ T cells, while the high-risk group demonstrated a higher proportion of M0 macrophages. Higher densities of CD4+ T cells are associated with a better prognosis. Thus, the low-risk group possessed a high density of CD4+ T cells, and longer survival may be explained by this finding (Ahlen Bergman et al., 2018; Schroeder et al., 2021). It has been suggested that significant infiltration of M0 macrophages in the tumor microenvironment may predict poor prognosis (Zheng et al., 2021). Liu et al. (2017) found that the number of M0 macrophages was inversely proportional to the survival time of lung cancer patients. In our study, we found that the high-risk group had a higher proportion of M0 macrophages than the low-risk group and that the high-risk patients had shorter survival, which is consistent with the findings above. In addition, an increase in the number of dendritic cells was associated with a good prognosis (Melaiu et al., 2020). Similarly, in our study, the low-risk group had an increased degree of dendritic cell infiltration and a better prognosis, which favorably supports this finding. Additionally, ssGSEA showed an increased proportion of neutrophils and plasmacytoid dendritic cells (pDCs) in the low-risk group. According to our analysis, 55% of patients with TP53 somatic mutations were in the high-risk group, while only 52% of patients in the low-risk group had these mutations. Cancer cells with TP53 mutations possess new tumor-promoting features, including higher invasion ability and metastatic capacity (Pitolli et al., 2019). Breast cancer patients with TP53 mutations have a poor prognosis (Zhang et al., 2021). Therefore, the high-risk group may be more likely to develop an immunosuppressive phenotype caused by TP53 mutations.

Chemotherapy resistance is a major hurdle in COAD treatment and is closely related to mortality risk. For chemotherapy-sensitive cancer patients, we can maximize the antitumor effect of chemotherapy drugs and confer a benefit from standard and scientific chemotherapy regimens. Our study demonstrated an increased sensitivity to paclitaxel, dasatinib, gefitinib, nilotinib, pazopanib, rapamycin, and sunitinib among high-risk patients. For patients with metastatic CRC, gefitinib combined with FOLFOX-4 pretreatment enhanced the efficacy of antitumor therapy (Kuo et al., 2005). Our findings may shed new light on suitable drug selection, helping to guide future oncology studies. However, the exact molecular mechanism by which chemotherapeutic drugs affect COAD patients requires further exploration and experimental validation. Therefore, the risk score may function as a diagnostic tool to assess the chemotherapeutic sensitivity of COAD patients, leading to a more individualized treatment approach.

In our study, we obtained four core molecular targets (DEFA5, CEACAM7, CLCA1, and ZG16) by joint analysis of the UniProt and PDB databases. Interestingly, DEFA5 has been identified as a critical biomarker of inflammatory bowel disease and plays a crucial anti-inflammatory role. In addition to serving as a novel immune response regulator, ZG16 may serve as a biomarker for immunotherapy. As an alternative application of the prognostic classifier, we explored the feasibility of searching for drug candidates based on a combination of the core molecular targets and structure-based approach. In the current study, we screened six small-molecule drugs with high affinity for core molecular targets. Among them, bongkrek acid functions as a selective activator of the peroxisome proliferator-activated receptor *γ* isoform. Esmolol is a well-established, fast β-blocker used to treat cardiac arrhythmias and hypertension. Norethisterone, a potent and widely available progestin, is recommended to treat endometrial hyperplasia. By enhancing the activity of bone morphogenetic protein 2, parbendazole induces osteogenic differentiation, exerting its osteoporosis-prevention effects. Although the specific mechanism of small-molecule compounds needs further experimental verification, our study preliminarily shows their potential in COAD treatment, guiding personalized cancer treatment.

Multiple approaches, datasets, and analyses were used to verify the accuracy and robustness of our model. However, several limitations exist that require further investigation. First, this study was conducted using only retrospective data from the TCGA database; thus, prospective studies are needed to explore its clinical value. Second, exploring the molecular mechanisms of glycolysis- and lactate-related genes in COAD progression will require more fundamental experiments. Finally, studies with larger sample sizes are necessary.

## Conclusion

In conclusion, we identified four COAD molecular subtypes with distinct prognoses and constructed a glycolysis- and lactate-related gene prognostic signature. The prognostic signature may function as a reliable indicator for prognosis prediction, immune cell infiltration and drug candidates and may be used to identify potential targets for accurate and efficient COAD therapy.

## Data Availability

The datasets presented in this study can be found in online repositories. The names of the repository/repositories and accession number(s) can be found in the article/Supplementary Material.

## Supplementary material

SUPPLEMENTARY FIGURE S1 KM survival curves of COAD patients stratified by age, gender, and stage.

SUPPLEMENTARY FIGURE S2 Validation of the glycolysis- and lactate-related gene prognostic signature in GSE17536 **(A)** and GSE39582 **(B)**.

SUPPLEMENTARY FIGURE S3
**(A,B)** An analysis of the glycolysis- and lactate-related gene prognostic signature using univariate **(A)** and multivariate **(B)** Cox regression. **(C,D)** DCA **(C)** and CIC **(D)** of the nomogram.

SUPPLEMENTARY FIGURE S4
**(A)** Relationships between the glycolysis- and lactate-related gene prognostic signature and CYT activity score. **(B)** Relationships between the glycolysis- and lactate-related gene prognostic signature and the TMB.

SUPPLEMENTARY FIGURE S5 Representative immunohistochemistry images of glycolysis- and lactate-related genes from the Human Protein Atlas database.
