# Supplementary material for: Identification of a glycolysis- and lactate-related gene signature for predicting prognosis, immune microenvironment, and drug candidates in colon adenocarcinoma
Source: Front Cell Dev Biol. 2022 Aug 23;10:971992. doi: 10.3389/fcell.2022.971992 (PMC9445192; doi:10.3389/fcell.2022.971992)
Supplement: Supplementary file 4 [file Presentation1.pdf]

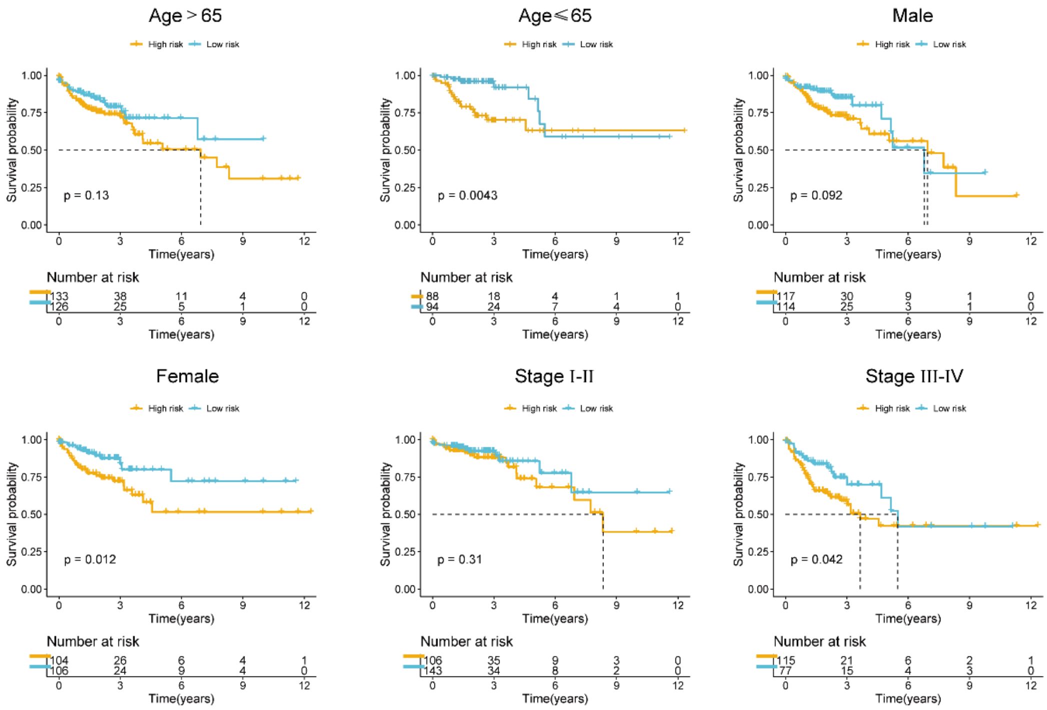

Supplementary Figure S1 | KM survival curves of COAD patients stratified by age, gender, and stage.

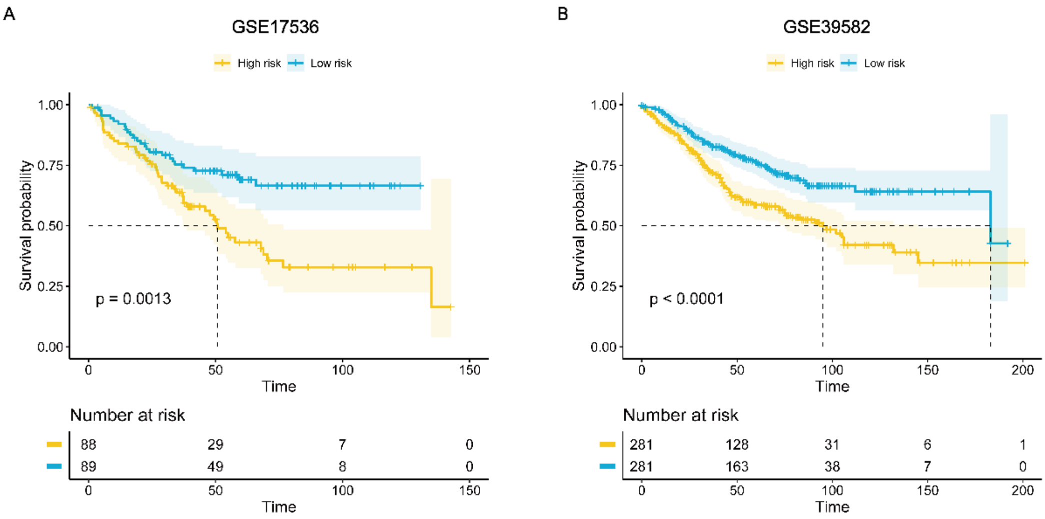

Supplementary Figure S2 | Validation of the glycolysis- and lactate-related gene prognostic signature in GSE17536 **(A)** and GSE39582 **(B)**.

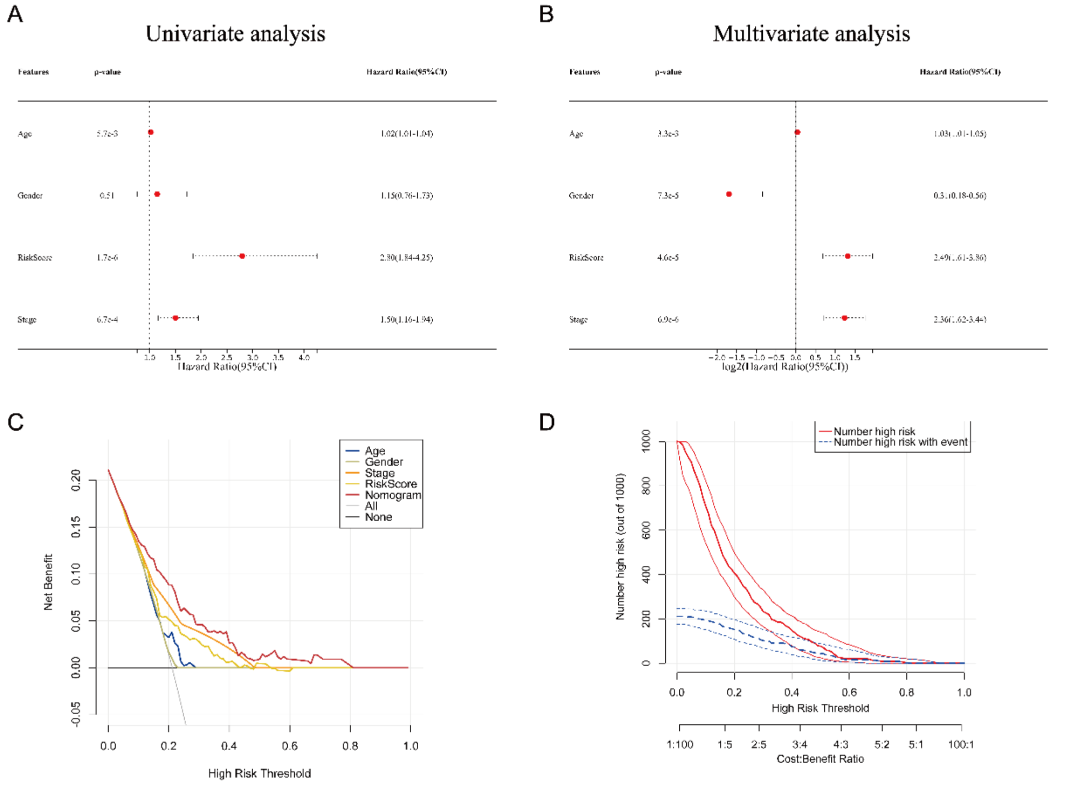

Supplementary Figure S3 | (A, B) An analysis of the glycolysis- and lactate-related gene prognostic signature using univariate **(A)** and multivariate **(B)** Cox regression. **(C, D)** DCA **(C)** and CIC **(D)** of the nomogram.

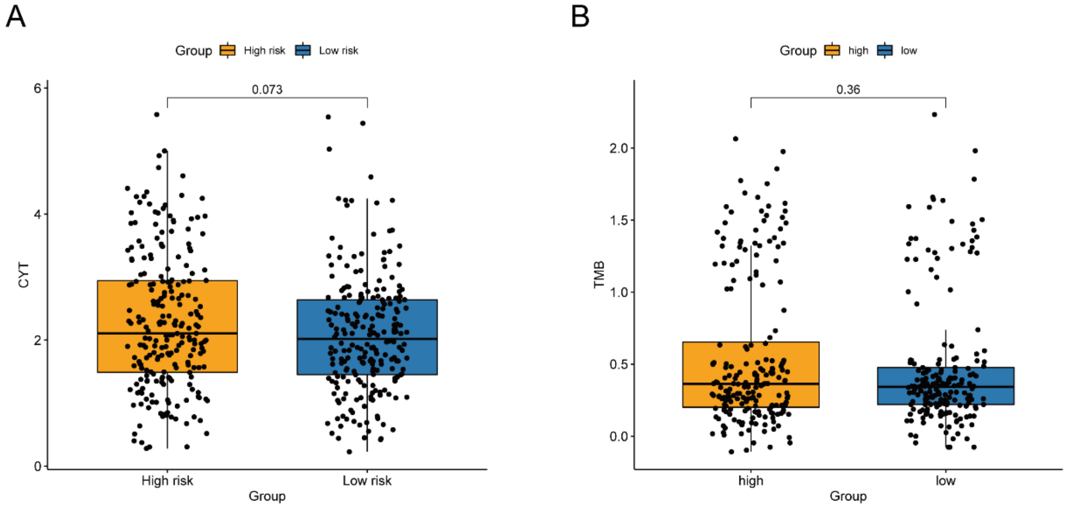

Supplementary Figure S4 | (A) Relationships between the glycolysis- and lactate-related gene prognostic signature and CYT activity score. **(B)** Relationships between the glycolysis- and lactate-related gene prognostic signature and the TMB.

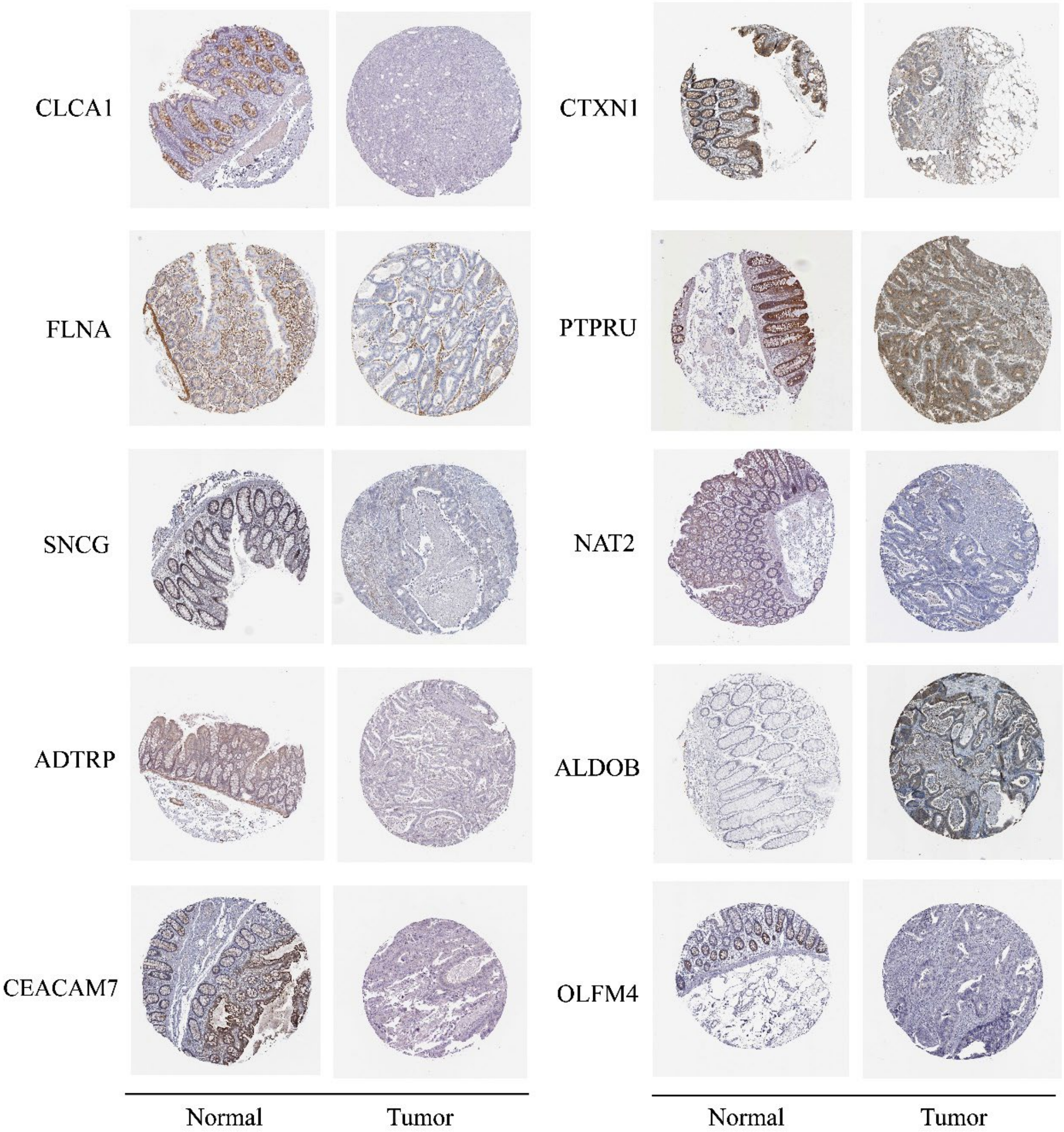

Supplementary Figure S5 | Representative immunohistochemistry images of glycolysis- and lactate-related genes from the Human Protein Atlas database.
