# Supplementary material for: Identification of a glycolysis- and lactate-related gene signature for predicting prognosis, immune microenvironment, and drug candidates in colon adenocarcinoma
Source: Front Cell Dev Biol. 2022 Aug 23;10:971992. doi: 10.3389/fcell.2022.971992 (PMC9445192; doi:10.3389/fcell.2022.971992)
Supplement: Supplementary file 5 [file Table2.DOCX]

library(NMF)

coad.log2fpkm.enengy <- data

ranks <- 2:10

estim.coad <- nmf(coad.log2fpkm.enengy,ranks, nrun=10,.options=list(keep.all=TRUE))

duplicated(colnames(coad.log2fpkm.enengy))

plot(estim.coad)

seed = 20

nmf.rank4 <- nmf(coad.log2fpkm.enengy,

rank = 4,

nrun=10,

seed = seed,

method = "brunet")

jco <- c("#2874C5","#EABF00","#C6524A","#868686")

index <- extractFeatures(nmf.rank4,"max")

sig.order <- unlist(index)

NMF.Exp.rank4 <- coad.log2fpkm.enengy[sig.order,]

NMF.Exp.rank4 <- na.omit(NMF.Exp.rank4) #sig.order有时候会有缺失值

group <- predict(nmf.rank4)

table(group)

consensusmap(nmf.rank4,

labRow = NA,

labCol = NA,

annCol = data.frame("cluster"=group[colnames(NMF.Exp.rank4)]),

annColors = list(cluster=c("1"=jco[1],"2"=jco[2],"3"=jco[3],"4"=jco[4])))

expr<-read.csv("")

rownames(expr)=expr[,1] #取出第一列

expr=expr[,-1]

expr<-log2(expr+1)

expr = avereps(expr[,-1],ID = expr$SYMBOL)

expr = expr[rowMeans(expr)>1,]

library(stringr)

tumor <- colnames(expr)[as.integer(substr(colnames(expr),13,14)) < 10]

normal <- colnames(expr)[as.integer(substr(colnames(expr),13,14)) >= 10]

tumor_sample <- expr[,tumor]

normal_sample <- expr[,normal]

exprSet_by_group <- cbind(tumor_sample,normal_sample)

group_list <- c(rep('tumor',ncol(tumor_sample)),rep('normal',ncol(normal_sample)))

data = exprSet_by_group

group_list = factor(group_list)

design <- model.matrix(~0+group_list)

rownames(design) = colnames(data)

colnames(design) <- levels(group_list)

DGElist <- DGEList( counts = data, group = group_list )

keep_gene <- rowSums( cpm(DGElist) > 1 ) >= 0 # 自定义

table(keep_gene)

DGElist <- DGElist[ keep_gene, , keep.lib.sizes = FALSE ]

DGElist <- calcNormFactors( DGElist )

v <- voom(DGElist, design, plot = TRUE, normalize = "quantile")

fit <- lmFit(v, design)

cont.matrix <- makeContrasts(contrasts = c('tumor-normal'), levels = design)

fit2 <- contrasts.fit(fit, cont.matrix)

fit2 <- eBayes(fit2)

nrDEG_limma_voom = topTable(fit2, coef = 'tumor-normal', n = Inf)

nrDEG_limma_voom = na.omit(nrDEG_limma_voom)

head(nrDEG_limma_voom)

P.Value = 0.05 # 自定义

foldChange= 0.75 # 自定义

nrDEG_limma_voom_signif = nrDEG_limma_voom[(nrDEG_limma_voom$P.Value < P.Value &

(nrDEG_limma_voom$logFC>foldChange | nrDEG_limma_voom$logFC<(-foldChange))),]

nrDEG_limma_voom_signif = nrDEG_limma_voom_signif[order(nrDEG_limma_voom_signif$logFC),]

write.csv(nrDEG_limma_voom,"zongchayi1.csv")

write.csv(nrDEG_limma_voom_signif,"chayi2.csv")

library(ggplot2)

data <- read.csv("")

rownames(data)=data[,1] #取出第一列

data=data[,-1]

ggplot(data,aes(logFC, -log10(adj.P.Val)))+

# 横向水平参考线：

geom_hline(yintercept = -log10(0.05), linetype = "dashed", color = "#999999")+

# 纵向垂直参考线：

geom_vline(xintercept = c(-1.2,1.2), linetype = "dashed", color = "#999999")+

# 散点图:

geom_point(aes(size=-log10(adj.P.Val), color= -log10(adj.P.Val)))+

# 指定颜色渐变模式：

scale_color_gradientn(values = seq(0,1,0.2),

colors = c("#39489f","#39bbec","#f9ed36","#f38466","#b81f25"))+

# 指定散点大小渐变模式：

scale_size_continuous(range = c(1,3))+

# 主题调整：

theme_bw()+

theme(panel.grid = element_blank())

data$label <- c(rownames(data)[1:2],rep(NA,(nrow(data)-2)))

ggplot(data,aes(logFC, -log10(adj.P.Val)))+

# 横向水平参考线：

geom_hline(yintercept = -log10(0.05), linetype = "dashed", color = "#999999")+

# 纵向垂直参考线：

geom_vline(xintercept = c(-1,1), linetype = "dashed", color = "#999999")+

# 散点图:

geom_point(aes(size=-log10(adj.P.Val), color= -log10(adj.P.Val)))+

# 指定颜色渐变模式：

scale_color_gradientn(values = seq(0,1,0.2),

colors = c("#39489f","#39bbec","#f9ed36","#f38466","#b81f25"))+

# 指定散点大小渐变模式：

scale_size_continuous(range = c(1,3))+

# 主题调整：

theme_bw()+

# 调整主题和图例位置：

theme(panel.grid = element_blank(),

legend.position = c(0.01,0.7),

legend.justification = c(0,1)

)+

# 设置部分图例不显示：

guides(col = guide_colourbar(title = "-Log10_q-value"),

size = "none")+

# 添加标签：

geom_text(aes(label=label, color = -log10(adj.P.Val)), size = 4, vjust = 1.5, hjust=1)+

# 修改坐标轴：

xlab("Log2FC")+

ylab("-Log10(FDR q-value)")

##划分

library(dplyr)

data <- read.csv("")

set.seed(123)

x <- nrow(data) %>% runif()

data <- transform(data,sample=order(x)) %>% arrange(sample)

data.1 <- data[1:(nrow(data)/2),]

data.2 <- data[((nrow(data)/2)+1):nrow(data),]

source("CIBERSORT.R")

if(F){

TME.results = CIBERSORT("LM22.txt",

"mianyi.txt" ,

perm = 1000,

QN = T)

save(TME.results,file = "ciber_CHOL.Rdata")

}

load("ciber_CHOL.Rdata")

TME.results[1:4,1:4]

re <- TME.results[,-(23:25)]

library(pheatmap)

k <- apply(re,2,function(x) {sum(x == 0) < nrow(TME.results)/2})

table(k)

re2 <- as.data.frame(t(re[,k]))

an = data.frame(group = Group,

row.names = colnames(exp))

pheatmap(re2,scale = "row",

show_colnames = F,

annotation_col = an,

color = colorRampPalette(c("navy", "white", "firebrick3"))(50))

library(RColorBrewer)

mypalette <- colorRampPalette(brewer.pal(8,"Set1"))

dat <- re %>% as.data.frame() %>%

rownames_to_column("Sample") %>%

gather(key = Cell_type,value = Proportion,-Sample)

ggplot(dat,aes(Sample,Proportion,fill = Cell_type)) +

geom_bar(stat = "identity") +

labs(fill = "Cell Type",x = "",y = "Estiamted Proportion") +

theme_bw() +

theme(axis.text.x = element_blank(),

axis.ticks.x = element_blank(),

legend.position = "bottom") +

scale_y_continuous(expand = c(0.01,0)) +

scale_fill_manual(values = mypalette(22))

ggplot(dat,aes(Cell_type,Proportion,fill = Cell_type)) +

geom_boxplot(outlier.shape = 21,color = "black") +

theme_bw() +

labs(x = "Cell Type", y = "Estimated Proportion") +

theme(axis.text.x = element_blank(),

axis.ticks.x = element_blank(),

legend.position = "bottom") +

scale_fill_manual(values = mypalette(22))

a = dat %>%

group_by(Cell_type) %>%

summarise(m = median(Proportion)) %>%

arrange(desc(m)) %>%

pull(Cell_type)

dat$Cell_type = factor(dat$Cell_type,levels = a)

ggplot(dat,aes(Cell_type,Proportion,fill = Cell_type)) +

geom_boxplot(outlier.shape = 21,color = "black") +

theme_bw() +

labs(x = "Cell Type", y = "Estimated Proportion") +

theme(axis.text.x = element_blank(),

axis.ticks.x = element_blank(),

legend.position = "bottom") +

scale_fill_manual(values = mypalette(22))

dat$Group = ifelse(as.numeric(str_sub(dat$Sample,13,14))<10,"High risk","Low risk")

library(ggpubr)

ggplot(dat,aes(Cell_type,Proportion,fill = Group)) +

geom_boxplot(outlier.shape = 21,color = "black") +

theme_bw() +

labs(x = "Cell Type", y = "Estimated Proportion") +

theme(legend.position = "top") +

theme(axis.text.x = element_text(angle=80,vjust = 0.5))+

scale_fill_manual(values = mypalette(22)[c(14,4)])+ stat_compare_means(aes(group = Group,label = ..p.signif..),method = "kruskal.test")

library(estimate)

OvarianCancerExpr <- system.file("extdata", "sample_input.txt", package="estimate")

read.table(OvarianCancerExpr)[1:4,1:4]

filterCommonGenes(input.f='mianyi.txt',#输入文件，为自己的表达矩阵

output.f="OV_10412genes.gct",#定义输出到工作目录的输出文件名，后缀为gct

id="GeneSymbol")#我们数据集的列名为GeneSymbol，因此这里选择拿GeneSymbol进行匹配

rt<-read.table("OV_10412genes.gct",

skip = 2,

header = TRUE,

sep = "\t")

View(rt)

estimateScore(input.ds = "OV_10412genes.gct", #刚才过滤得到的输入文件

output.ds="estimateScore.gct", #输出的输出文件

platform="illumina") #注意平台，如果为TCGA或者测序数据则选择illumina

scores=read.table("estimateScore.gct",#读取文件

skip = 2,#删除前2行

header = T)#第一行为列名

View(scores)

rownames(scores)=scores[,1]#取第一列为行名

scores=t(scores[,3:ncol(scores)])#取3列到最后1列的数据并进行数据转置

View(scores)

TumorPurity = cos(0.6049872018+0.0001467884 * scores[,3])

head(TumorPurity)

write.csv(scores,"scores.csv")

write.csv(TumorPurity,"TumorPurity.csv")

mydata<-read.csv("scores1.csv")

p <- ggboxplot(mydata, x = "Group", y = "TumorPurity",

color = "Group", palette = c("#FFA911", "#377EB8"),

add = "jitter")

p + stat_compare_means()

library(pRRophetic)

library(ggplot2)

esprSet<-read.csv("gaodi.csv")

rownames(esprSet)=esprSet[,1] #取出第一列

esprSet=esprSet[,-1]

expr=as.matrix(esprSet)

predictedType<-pRRopheticPredict(expr,"Gefitinib",selection = 1)

write.csv(predictedType,"19.csv")

mydata<-read.csv("1.csv")

p <- ggboxplot(mydata, x = "Group", y = "Paclitaxel",

fill = "Group", palette = c("#FFA911", "#377EB8"),

)

p + stat_compare_means()+theme(plot.title = element_text(hjust = 0.5,size = 20, face = "bold"),axis.text=element_text(size=12,face = "bold"),axis.title.x=element_text(size=14),axis.title.y=element_text(size=14))

p <-ggplot(data = mydata,aes(y = Gemcitabine, x = Group,fill=Group))+

geom_violin()+

theme_bw()+

ylab('Gemcitabine senstivity (IC50)') +theme(plot.title = element_text(hjust = 0.5,size = 20, face = "bold"),axis.text=element_text(size=12,face = "bold"),axis.title.x=element_text(size=14),axis.title.y=element_text(size=14))+

xlab('Group')

p + stat_compare_means(vjust = 10,

tip.length = 3,

bracket.size = 1,

step.increase = 0,)

p <- ggviolin(mydata, x = "Group", y = "Paclitaxel",

fill = "Group", add = "boxplot",add.params = list(fill="white"),palette = c("#FFA911", "#377EB8"),

)

comparisons <- list(c("High risk", "Low risk"))

p + stat_compare_means(comparisons=comparisons)+ylab('Lapatinib senstivity (IC50)')+theme(plot.title = element_text(hjust = 0.5,size = 20, face = "bold"),axis.text=element_text(size=12,face = "bold"),axis.title.x=element_text(size=14),axis.title.y=element_text(size=14))

library(ggpubr)

library(ggplot2)

p <- ggboxplot(dat, x = "Cell_type", y = "Proportion",

color = "Group", palette = c("#FFA911", "#377EB8"),

)

p+labs(x = "", y = "Score") +theme(axis.text.x = element_text(angle=80,vjust = 0.5))+stat_compare_means(aes(group = Group,label = ..p.signif..),method = "kruskal.test")

library("tidyverse")

data <- read.csv("cell.csv")

rownames(data)=data[,1] #取出第一列

data=data[,-1]

data=log2(data+1)

dat=data

write.csv(dat,"hla1.csv")

re=data

dat <- re %>% as.data.frame() %>%

rownames_to_column("Sample") %>%

gather(key = Cell_type,value = Proportion,-Sample)

dat$Group = ifelse(as.numeric(str_sub(dat$Sample,13,14))<10,"High risk","Low risk")

ggplot(dat,aes(Cell_type,Proportion,fill = Group)) +

geom_boxplot(outlier.shape = 21,color = "black") +

theme_bw() +

labs(x = "", y = "Gene expression") +

theme(legend.position = "top") +

theme(axis.text.x = element_text(angle=80,vjust = 0.5))+

scale_fill_manual(values = mypalette(22)[c(14,4)])+ stat_compare_means(aes(group = Group,label = ..p.signif..),method = "kruskal.test")
