# Supplementary material for: Identification of a glycolysis- and lactate-related gene signature for predicting prognosis, immune microenvironment, and drug candidates in colon adenocarcinoma
Source: Front Cell Dev Biol. 2022 Aug 23;10:971992. doi: 10.3389/fcell.2022.971992 (PMC9445192; doi:10.3389/fcell.2022.971992)
Supplement: Supplementary file 7 [file Table3.DOCX]

The download link of mutation profiles of TCGA-COAD patients was

https://www.jianguoyun.com/p/DZiu0O0Q8_m5ChiUhbgEIAA

The download link of RNA sequence transcriptome data of TCGA-COAD patients was

<https://www.jianguoyun.com/p/DaDZ-VIQ8_m5ChiahbgEIAA>
